# Organic Bioelectronics in Microphysiological Systems: Bridging the Gap Between Biological Systems and Electronic Technologies

**DOI:** 10.3390/bios15040253

**Published:** 2025-04-16

**Authors:** Pauline Coquart, Andrea El Haddad, Dimitrios A. Koutsouras, Johanna Bolander

**Affiliations:** 1 Research Unit ‘Soft Matter and Biophysics’, Department ‘Physics and Astronomy’, KU Leuven, B-3000 Leuven, Belgium; pauline.coquart@imec.be; 2 IMEC, Kapeldreef 75, B-3001 Leuven, Belgium; andrea.elhaddad@imec.be; 3 Research Unit ’Assiocated Division ESAT-INSYS (INSYS), Integrated Systems’, Department ‘Electrical Engineering (ESAT)’, KU Leuven, B-3000 Leuven, Belgium; 4 IMEC NL, 5656 AE Eindhoven, The Netherlands; 5 Department of Electronic & Electrical Engineering, University of Bath, Claverton Down, Bath BA2 7AY, UK; 6 Centre for Bioengineering & Biomedical Technologies (CBio), University of Bath, Claverton Down, Bath BA2 7AY, UK; 7 Berlin Institute of Health Center for Regenerative Therapied (BCRT), Berlin Institute of Health at Charité—Universitätmedizin Berlin, 13353 Berlin, Germany; 8 Julius Wolff Institute for Biomechanics and Musculoskeletal Regeneration, Berlin Institute of Health at Charité—Universitätmedizin Berlin, 13353 Berlin, Germany

**Keywords:** organic bioelectronics, conducting polymers, microphysiological systems, biology–technology interface

## Abstract

The growing burden of degenerative, cardiovascular, neurodegenerative, and cancerous diseases necessitates innovative approaches to improve our pathophysiological understanding and ability to modulate biological processes. Organic bioelectronics has emerged as a powerful tool in this pursuit, offering a unique ability to interact with biology due to the mixed ionic–electronic conduction and tissue-mimetic mechanical properties of conducting polymers (CPs). These materials enable seamless integration with biological systems across different levels of complexity, from monolayers to complex 3D models, microfluidic chips, and even clinical applications. CPs can be processed into diverse formats, including thin films, hydrogels, 3D scaffolds, and electrospun fibers, allowing the fabrication of advanced bioelectronic devices such as multi-electrode arrays, transistors (EGOFETs, OECTs), ion pumps, and photoactuators. This review examines the integration of CP-based bioelectronics in vivo and in in vitro microphysiological systems, focusing on their ability to monitor key biological events, including electrical activity, metabolic changes, and biomarker concentrations, as well as their potential for electrical, mechanical, and chemical stimulation. We highlight the versatility and biocompatibility of CPs and their role in advancing personalized medicine and regenerative therapies and discuss future directions for organic bioelectronics to bridge the gap between biological systems and electronic technologies.

## 1. Introduction

The global population faces significant health challenges due to the increasing burden of a range of diseases, including fibrotic and degenerative disorders, cardiovascular diseases, infectious diseases, neurodegenerative conditions, and cancers, all of which collectively contribute to substantial morbidity and mortality [1]. However, extensive research efforts are focused on unraveling the underlying pathophysiology and developing groundbreaking therapies to improve outcomes and reduce their global burden.

To address these health challenges, researchers have developed various approaches to monitor, characterize, and modulate biological events, advancing both our understanding and treatment strategies. These approaches include (i) the direct monitoring and modulation of the human physiology through wearable and implantable electronics such as pacemakers, glucose sensors, electroencephalograms, or electrocardiograms, enabling real-time prevention and therapeutic interventions; (ii) investigating disease mechanisms in vivo using animal models, which remain the gold standard in studying physiological and pathological processes in a whole-organism context; and (iii) developing in vitro models that replicate key properties of organs or tissues, with microphysiological systems (MPSs) emerging as the next-generation alternative to in vivo systems in understanding complex biological processes.

**Figure 1:**
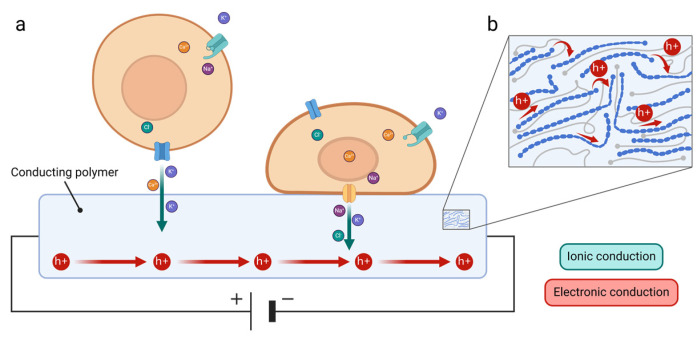
Mixed ionic and electronic conduction of conducting polymers. Organic mixed ionic–electronic conductors (OMIECs) can simultaneously transport (**a**) ions through the polymer volume and charge carriers along the polymer backbone through (**b**) hopping or band-like transport mechanisms.

Many biological events, such as heartbeats, neural network communication, or tissue regeneration, are driven by ionic currents [2]. Therefore, understanding and being able to control these ionic processes provides critical insights into how these events are initiated and regulated. Organic bioelectronics emerges as a powerful tool in this task [3,4,5] due to two key properties, namely (i) their mixed ionic and electronic conduction, which enables the transduction of ionic into electronic signals and serves as a seamless communication pathway between biology and technology (Figure 1), and (ii) the organic’s mechanical properties, which allow integration with biological systems by mimicking the mechanical characteristics of tissues and organs and enable proximity to the source of biological signals for precise transduction. These features significantly improve the biology–electronic interface compared to traditional inorganic materials.

Among organic materials, conducting polymers (CPs) have gained increasing interest over the last few decades due to their strong electrical properties, intrinsic electrochemical activity, and mechanical flexibility [6]. Their versatility has enabled their use in fabricating advanced bioelectronic devices including multi-electrode arrays (MEAs), electrochemical transistors such as electrolyte-gated organic field-effect transistors (EGOFETs) and organic electrochemical transistors (OECTs), organic electronic ion pumps (OEIP), and photoactuators. CPs have also been employed as coatings to enhance the performance of existing devices composed of conventional materials, improving signal transduction and overall data quality. Unlike metals or emerging nanomaterials like graphene [7] or MXenes [8], which require additional biofunctionalization steps to enhance their biocompatibility, CPs inherently support direct biological integration while keeping their advantageous electrical properties, enabling the development of innovative and biocompatible bioelectronic devices.

This synergy between soft, biocompatible technology and biological systems offers promising potential in advancing areas such as personalized medicine, regenerative therapies, or biohybrid systems, acting as a driving force for researchers to explore novel applications of organic bioelectronics.

In this review, we explore how and when organic bioelectronics can be integrated with biological systems to study key biological events driven by ionic activity in vivo and in in vitro MPSs. Specifically, we discuss the unique properties of CPs that make them suitable for interfacing with biology, and we present the advantages that they offer in developing bioelectronic devices such as electrodes, transistors, ion pumps, and photoactuators. Furthermore, this review provides a comprehensive overview of the current state of the art, discussing how CPs have been leveraged in both in vivo and in vitro models, identifying current challenges and offering perspectives on the development of next-generation devices.

## 2. Biology to Be Studied with Organic Bioelectronics

Biological signals are represented by various molecular entities of different compositions, sizes, or functions, ranging from ions and neurotransmitters to DNA and proteins, and convey information within biological systems and networks. This useful information can be used to understand the underlying physiological mechanisms of specific biological events or systems, which subsequently provides an opportunity to steer and/or stimulate these in specific directions.

Organic bioelectronic devices aim to interface biology and related signals with conventional electronics that rely exclusively on electrons as the dominant charge carrier [4]. Indeed, they possess mixed ionic and electronic conduction mechanisms that position them as transducers of biological to electronic signals, acting as a communication bridge across the biology–technology gap [3].

Furthermore, the high coupling efficiency between electronics and biology is due to the capacity of organic electronics to reduce the physical and mechanical mismatch with biology. Adapting their interface in terms of shape, size, flexibility, and elasticity to comply with different biological architectures, as well as promoting their biocompatibility and stability over extended periods, further enhances their potential to stimulate, monitor, and interface with the majority of biological systems [9]. In particular, organic bioelectronic devices are used to study or stimulate different areas of biology, including neuroscience, cardiology, cellular biology, and wound healing [10]. Interfacing carbon-based electronics materials with living systems ranging from organs, cells, and cell membranes to 3D in vitro models and MPSs provides insights into many research fields, including electrophysiology, tissue engineering, drug release, biosensing, and molecular bioelectronics [11].

**Figure 2:**
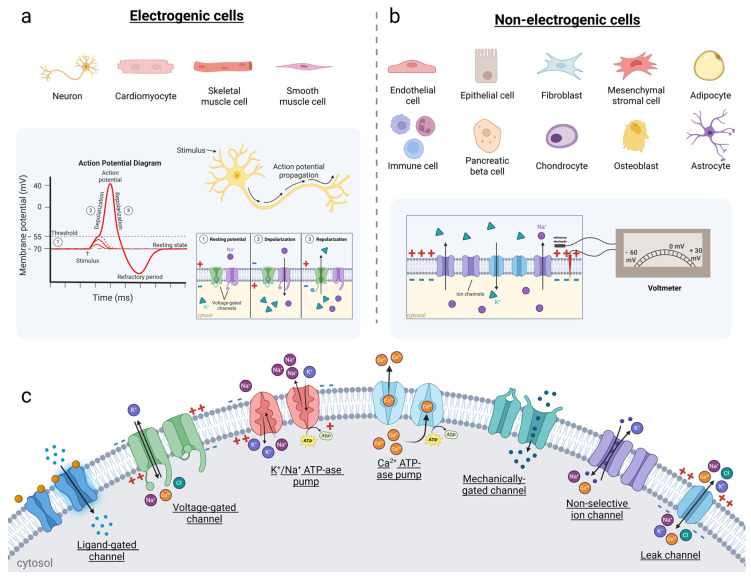
Summary of ion channel and pump activities in different cell types. Overview of the (**a**) electrogenic and (**b**) non-electrogenic cells and illustration of the roles of (**c**) ion channels and pumps in generating and propagating action potentials or maintaining the osmotic balance across the cellular membrane.

Organic bioelectronics can interface with two types of cells: (1) electrogenic cells, which actively generate action potentials (APs) and electrical signals, typically through ion channels and membrane potentials, and play central roles in electrical signaling in the body, and (2) non-electrogenic cells, which do not actively generate or propagate APs but respond to stimulation from electrogenic cells and possess ion channels to maintain cellular homeostasis and perform metabolic or structural roles (Figure 2). By improving the interface between biology and electronics, organic bioelectronics enable the monitoring and stimulation of both electrogenic and non-electrogenic cells’ transmembrane potentials and associated pathways to study the initiation and regulation of a wide range of biological events and systems, thus facilitating the understanding of underlying mechanisms and pathologies.

### 2.1. Transmembrane Potential

Every cell of the body has electrical potentials across its membrane, which play important roles in regulating various biological processes. The resting membrane potential is a continuous and dynamic process that maintains a steady ionic balance of several important ions, including sodium (Na^+^), potassium (K^+^), chloride (Cl^−^), and calcium (Ca^2+^), across the cellular membrane [12]. The transport of ions across the selectively permeable membrane is established and regulated by ion pumps and channels, gap junctions, and specialized receptor/transporter molecules.

Ion channels are highly selective pores, allowing the passive transport of ions through the cellular membrane according to their concentration gradient (Figure 2c). Under appropriate stimulation, they change their conformation to allow ion flux. Ion channels can open and close in response to changes in voltage or membrane potential (voltage-gated Na^+^, K^+^, Ca^2+^ channels), the binding of ligands such as neurotransmitters (transmitter-, ion-, nucleotide-gated channels), or even light (light-gated channels) [13]. Ion pumps actively transport ions against their concentration gradients. Pumps like Na^+^/K^+^-ATPase generate an electrochemical gradient that maintains ionic gradients across the membrane by actively transporting Na^+^ and K^+^ out of and into the cells, respectively [14] (Figure 2c).

Whenever a stimulus (mechanical, chemical, or electrical) is applied over the cell membrane at the resting membrane potential, the polarity of the membrane is modified, initiating and/or regulating numerous biological events. Given that organic bioelectronic devices possess mixed ionic and electronic conduction, they can monitor the transmembrane potential by translating ionic movements into electrical signals, thereby providing valuable information on the initiation and/or progression of these biological processes. They can even influence the membrane potential, therefore becoming an effective tool for the study of biological systems.

### 2.2. Electrogenic Cells

Electrogenic cells, such as nerve axons and muscle fibers (cardiac and skeletal), are a specific cell type that exhibit the property of excitability, by which they can transmit unique electrical signals along their membranes.

Their resting membrane potential is highly negative (typically between −60 and −90 mV), and they possess high densities of voltage-gated Na^+^, K^+^, and sometimes Ca^2+^ channels in their membranes. Changes in membrane potential lead to the rapid reversal of membrane polarity, characterized by the rapid depolarization and repolarization of the cell membrane involving voltage-gated Na^+^ and K^+^ channels [15] (Figure 2a). The rapid change in electrochemical impulse generates an AP and transmits signals along the cell membrane, indicating the start of cellular activity [15].

The resting membrane potential and APs play a critical role in various cellular and physiological functions, such as neuronal communication, as membrane depolarization triggers nearby voltage-gated Na^+^ channels to open, propagating the AP down the axon. It also influences muscle contraction as the activation of K^+^ ion channels induces membrane hyperpolarization, resulting in decreased Ca^2+^ influx, leading to the relaxation of vascular smooth muscle cells [2]. They are also involved in other functions, such as hormone secretion, tissue formation and regeneration, ion transport and homeostasis, immune cell activation and cytokine release, barrier function, and cell apoptosis, among others.

#### 2.2.1. AP Propagation

Electrogenic cells that generate and propagate APs through voltage-gated Na^+^, K^+^, and Ca^2+^ channels include neurons, cardiomyocytes, skeletal muscle cells, smooth muscle cells, and pancreatic beta cells [16]. The ability to record, stimulate, or modulate their electrical signals provides a unique opportunity to improve our understanding regarding their activity, function, and communication (e.g., how the brain encodes information and controls behavior [17]).

Organic bioelectronic devices can be utilized for the extracellular electrophysiological recording of APs or intracellular recordings of voltage changes in response to stimuli. Even if the electrophysiological signals (i.e., ionic potential fluctuations) are weak and therefore difficult to record, organic bioelectronics are able to amplify them directly at the electrode site by implementing voltage-to-voltage conversion at the front-end recording pixel [18]. Thus, the quality of the data collected with amplifiers is improved, compared to that without them, enabling the monitoring of cellular electrical activity for further study.

The monitoring and recording of cellular electrical activity are performed through implantable and external devices. Indeed, CP-based electrodes can be designed with soft and flexible characteristics, which make them ideal for chronic implantation in the brain and the recording of the activity of hundreds of single neurons [19]. They can also perform non-invasive electroencephalography (EEG) recording to investigate the frequency of brainwaves and detect different states of mental functions [20] or to build non-invasive on-skin electrodes for continuous and real-time electrocardiography [21].

Besides recording, these devices can be implemented to electrically stimulate cells by applying electrical currents to excite neurons or muscle cells for functional studies. CP-based electrodes [22] or coated carbon fibers [23] are used to deliver electrical stimulation to specific areas of the brain or spinal cord. This is performed to modulate neural activity, which is useful in treating neurological disorders (e.g., Parkinson’s disease or spinal cord injury). Electrical stimulation through three-dimensional conductive scaffolds can also support adhesion, proliferation, and myocardial differentiation studies [24]. In addition, it can be utilized to support cell electrical signaling and facilitate the propagation of electrical currents across scarred tissues, improving cardiac function [25] or preventing arrhythmia [26].

#### 2.2.2. Ca^2+^-Dependent Neurotransmitter Release at Synaptic Terminals

Organic bioelectronics include sensitive sensors with the ability to detect the release of neurotransmitters like glutamate, GABA, and acetylcholine in response to APs. In neurons, voltage-gated Ca^2+^ channels at synaptic terminals allow the entry of Ca^2+^, which triggers the vesicular release of neurotransmitters at synapses for communication with other neurons or target cells [27]. Neurochemical sensing with CPs can measure subtle neurotransmitter releases and changes. For example, CP-based sensors can detect dopamine release in the rat brain within a concentration range of 30 nM to 0.1 mM [28], thus providing insights into brain chemistry during different states or in response to stimuli.

#### 2.2.3. Intracellular Ion Channel Modulation

Organic bioelectronics are also able to modulate ion channel activation through optogenetic stimulation. This technique combines an organic electronic device with optical fibers to activate engineered light-sensitive Ca^2+^ channels or receptors, allowing the precise control of cellular activity. This technique has been used to stimulate rats’ peripheral nerves and brains through the stimulation of neurons, including genetically engineered light-gated ion channels [29]. In addition to controlling neurons’ membrane ionic conductivity, cell membrane capacitance has also been manipulated, thus modifying neuronal activity and enabling the long-lasting modulation of neuronal excitability [30]. Optogenetics is of particular interest as it allows for the highly precise spatiotemporal interrogation of specific cell types or biological processes [31].

Organic bioelectronic technologies have shown to enable precise control over the transmembrane potential of electrogenic cells, facilitating the monitoring and modulation of AP propagation, Ca^2+^-dependent neurotransmitter release, and ion channel activity. This capability can significantly aid in deepening our understanding regarding cellular excitability and signaling. In addition, the technology can be further extended to interface with non-electrogenic cells, expanding the scope of organic bioelectronics in diverse biological contexts.

### 2.3. Non-Electrogenic Cells

Non-electrogenic cells encompass a wide range of cell types that possess ion channels and pumps to regulate ion homeostasis and signaling by maintaining a resting membrane potential (typically less negative than that of electrogenic cells, around −30 to −60 mV), but they do not generate APs (Figure 2b). An endogenous electric field is present at the organelle, cellular, tissue, and organism levels, arising from gradients of charge carriers that are generated through the diffusion or active transport of charges across membranes.

Ion channels and pumps in non-electrogenic cells play a critical role in metabolic functions such as osmotic balance, nutrient transport, and volume regulation [32]. They respond to osmotic changes or mechanical forces (e.g., stress or pressure), inducing changes in cell state, gene expression, matrix production, and cell shape, rather than electrical activity. In the case of mechanical signaling, the responses are mediated through integrins, focal adhesions, and cytoskeletal interactions [33].

Ion channels are also involved in other non-excitable roles, such as biomolecule secretion, the immune response, tissue maintenance, and regeneration upon trauma, among others. For example, the Ca^2+^ signaling pathways play a significant role in various cellular processes, such as cell proliferation and differentiation, matrix production, cell migration, and intracellular signaling pathways (e.g., the activation of enzymes, gene expression, etc.) [34].

Non-electrogenic cells interact with organic bioelectronics primarily through their ionic fluxes, membrane potentials, and secretion of biochemical signals, rather than through large electrical potentials. Unlike inorganic electronics, organic electronic devices possess the ability to detect these subtle physiological processes in non-excitable cells and can even influence cellular responses under specific conditions.

#### 2.3.1. Ion Transport, Signaling, and Homeostasis

Many non-electrogenic cells, such as epithelial and endothelial cells, fibroblasts, mesenchymal stem cells, adipocytes, immune cells, and chondrocytes, undergo significant ion fluxes associated with physiological activities like metabolism and ion transport. They maintain homeostasis and respond to environmental cues by regulating ions such as K^+^, Na^+^, Ca^2+^, and Cl^−^. However, the signals generated by non-electrogenic cells are subtle as the majority of cell–cell communication mechanisms use extremely low frequencies, in the millihertz range [35].

Organic bioelectronic devices are particularly interesting sensors to access these weak bioelectrical signals. They are low-noise devices due to their low interfacial resistance with liquids, which minimizes thermal noise and provides a low-impedance path for slow-varying or long-lasting signals [36]. Thus, their low intrinsic thermal noise maximizes the signal-to-noise ratio (SNR) and makes organic bioelectronics an ideal tool to detect subtle ion fluxes and long-lasting oscillations generated by non-excitable cells.

Therefore, CPs can be integrated with ion-selective membranes to perform real-time measurements of ion concentrations. For example, organic-based electronics have been combined with Na^+^- and K^+^-selective membranes and incorporated with a microfluidic device to perform the real-time and reversible sensing of Na^+^ and K^+^ ions [37]. This could be used to provide insights into the cell status and responses to stimuli. Similarly, ion-selective polymeric membranes have been deposited on textile fibers functionalized with CP-based active films, with the aim of monitoring electrolytes in sweat using wearable sensors [38].

#### 2.3.2. Metabolic Pathways and Redox Signaling

Cellular metabolism is fundamental to life. It refers to the chemical processes that occur within the cells that generate the metabolic energy required for the majority of cellular activities. Cell metabolism is organized into a network of metabolic pathways, which generate energy in the form of adenosine triphosphate (ATP) through two major pathways: glycolysis (aerobic and anaerobic) and oxidative phosphorylation [39].

A key source of energy is glycolysis, where glucose is broken down into pyruvate. Under anaerobic conditions, pyruvate is converted to lactate, while, under aerobic conditions, it is shuttled into the mitochondrial citric acid cycle, where it is oxidized, producing ATP and reactive oxygen species (ROS) [40] as by-products. Many of the metabolites of glycolysis and the citric acid cycle can enter anabolic pathways, which generate NADPH and the building blocks needed for the generation of glycogen, lipid, nucleotide, and protein synthesis [41]. They can also enter catabolic processes, generating reduced nicotinamide adenine dinucleotide (NADH) and reduced flavin adenine dinucleotide (FADH2), which are used by the electron transport chain for the coordinated movement of electrons through a series of redox reactions across the inner mitochondrial membrane, releasing energy [42].

With their superior electrical conductivity, high electron affinity, and redox activity, CPs are suitable materials for electrochemical sensing. Indeed, through functionalization with enzymes or redox-active species [43] that selectively react with the molecule of interest (i.e., glucose, lactate, NADH, ROS, or ATP), an electrochemical reaction can be induced. This leads to a measurable current, voltage, or resistance change in the polymer, signaling the presence of the molecule. Thus, cellular metabolism in tissues like the liver or muscle can be monitored in real time [44].

CPs have been mainly used for glucose [45] and lactate [46] detection as they are key parameters to assess patients’ health conditions. Glucose oxidase (GOx) is a commonly used enzyme for glucose sensing, while lactate oxidase (LOx) and lactate dehydrogenase are used for lactate sensing. These enzymes catalyze the oxidation of glucose and lactate, respectively, in the presence of dissolved oxygen. This oxidation reaction produces hydrogen peroxide and leads to an electron flow that is measured to quantify the specific molecular amount [47].

As the blood glucose level in sweat corresponds to the actual blood glucose level, wearable electrochemical biosensors based on organic bioelectronics have been rapidly developed as a sensitive sensing technology for this application [47]. For both molecules, the developed electrochemical biosensors are mainly based on hydrogels. Hydrogel patches have been combined with polymer-based multilayered sweat glucose sensors to capture, collect, and quantify the analyte in different parts of the body with a high sweat gland density [48]. CPs have also been integrated directly into hydrogels to perform lactate [49] and glucose [50] screening in biofluids. Polymer-coated scaffolds can also serve as 3D electrochemical sensor integrated into 3D cell cultures [51].

Similarly, organic bioelectronics can monitor other molecules involved in metabolic pathways, such as NADH [52], the pH, or tyrosine [53].

#### 2.3.3. Tight Junction Integrity and Paracellular Transport

Paracellular transport and tight junction formation and disruption are typical mechanisms used to assess the properties of tissue barriers, such as the blood–brain barrier, alveolar capillary barrier, intestinal epithelial barrier, or skin epidermis.

Organic bioelectronics offer the possibility to monitor the transepithelial–transendothelial electrical resistance (TEER) of these barriers. They therefore provide valuable information on barrier integrity and permeability in a non-optic, fast, and non-invasive fashion and offer critical insights into the health, functionality, and disease status of tissues and organs [54]. Measuring the TEER with organic electrodes allows the early indication of drug performance by, for example, mapping local and real-time changes in epithelial barrier function at the air–liquid interface [55]. This type of measurement can also distinguish several cell types (e.g., epithelial or cancerous), making it a useful tool for the study of pathological processes such as cancer invasion of the epithelial monolayer [56].

Electrochemical impedance spectroscopy (EIS) complements TEER by providing a more detailed analysis of barrier or tissue properties. While TEER measures resistance, EIS allows the decomposition of electrical components such as capacitance, resistance, and reactance at various frequencies, offering insights into the dynamic behavior of tight junctions, cellular membranes, and their interfaces. This broader frequency-dependent analysis can reveal subtle changes in the barrier’s properties and interactions, which may not be captured with TEER alone.

Controlled and targeted drug delivery through the epithelial barrier can also be achieved with electrical stimulation. Indeed, voltage pulses (i.e., electroporation) can temporarily modulate the epithelial barrier’s permeability, allowing the passage of drugs [57].

#### 2.3.4. Cellular Functions

A unique feature of organic bioelectronics is that they can provide both electrical cues and structural support to cells to promote their cellular activities and tissue regeneration. The polymer’s electrical characteristics, in conjunction with a high level of biomimicry, open up new avenues in the development of advanced tissue models [58,59].

Constructs such as films, nanofibers, hydrogels, and 3D porous scaffolds are extensively used in bioengineering to emulate and regenerate living tissues. Combining them with electroactive CPs offers the possibility to promote cell activity and tissue formation, as well as monitor and stimulate cellular functions in vivo and in vitro, paving the way for new applications in tissue engineering [60,61,62].

Conducting 3D constructs integrate sensing capabilities by performing the real-time and non-invasive monitoring of the local electrical activity. Indeed, in the form of polymer-based cell-embedded scaffolds [63] or membranes separating compartmentalized cell cultures [64], organic bioelectronics enable the in situ recording of cell growth and function through EIS measurements. Furthermore, impedance spectroscopy allows us to distinguish various cell electrical signatures, as different tissue types result in different impedance spectra.

Organic bioelectronics can also be used to stimulate and enhance cellular functions such as migration, proliferation, differentiation, matrix production, and tissue remodeling by electrical stimulation. It has been shown that electrical stimulation drives Ca^2+^/ATP oscillations, which are essential for prechondrogenic condensation and subsequent chondrogenesis [65]. Similarly, three-dimensional cell-embedded porous conducting scaffolds can promote the cell adhesion and chondrogenesis of chondroprogenitor cells [66]. Electrical stimulation on adipose stem cells embedded in a scaffold has also been shown to enhance their growth and differentiation toward smooth muscle cells [67]. Furthermore, conducting hydrogels can be used to stimulate wound healing. By positioning the hydrogel on the wound and applying targeted electrical stimulation, enhanced cellular migration and angiogenesis can be achieved, promoting healing [68].

Although non-electrogenic cells cannot generate APs, organic bioelectronics provide a powerful tool for the study of a wide array of physiological processes that are crucial in their signaling and function, due to their ability to capture and transduce critical cellular processes with a low signal-to-noise ratio. Through the monitoring and modulation of ion transport, metabolic processes, barrier integrity, and cellular functions, organic bioelectronics enable a deeper understanding of the dynamic events governing non-electrogenic cells.

#### 2.3.5. Biocompatibility

When studying biological events or systems with organic bioelectronics, it is essential to ensure that the measurement and device itself does not interfere with or harm the biological system to be studied. A primary consideration for this is biocompatibility, as the interaction between technology and biology must not disrupt cellular functions or damage physiological processes.

Assessing the biocompatibility of materials used in organic bioelectronics is essential to prevent adverse biological responses, to ensure reliable experimental outcomes, and to maintain stable conditions for the study of living systems. Organic materials are known for their remarkable biocompatibility. However, biocompatibility is a theoretical concept suggesting compatibility across all biological environments, whereas, in reality, it depends on factors such as the biological system being studied and the testing methods used. These methods vary depending on whether the focus is on the cellular level or the entire biological system’s interaction with the device.

At the cellular level, biocompatibility is assessed through cell viability and cytotoxicity. Cell viability measures the number of healthy (viable) cells versus non-viable cells, while cytotoxicity evaluates whether a material induces cell death. These parameters can be studied using, for example, fluorogenic dyes, which differentiate between living and dead cells through distinct staining to assess cell viability, or ATP assays, which detect cytotoxic effects by measuring ATP reduction [69,70]. For example, a study evaluated the cytotoxicity of various PEDOT-based CPs with human neuroblastoma using an ATP assay. It was found that the ATP levels, proportional to the number of living cells, were comparable to those of control samples, indicating that these materials did not induce cytotoxic responses within this condition [71].

At the whole-system level, biocompatibility involves studying interactions between the device and the living system while also identifying potential adverse reactions over time. Regulatory bodies such as the European Medicines Agency (EMA), the U.S. Food and Drug Administration (FDA), and the International Organization for Standardization (ISO 10993 [72]) have set guidelines to ensure that medical devices meet biocompatibility standards before reaching the market. These evaluations include cytotoxicity testing at the cellular level, as well as assessments of general tissue sensitization, irritation, and immunotoxicology, considering the full biological system [69,70,73]. For example, irritation tests assess the potential of a material to cause irritation at sites associated with the implantation or contact site, such as eye irritation studies in animal or human models. These tests typically involve a one-second exposure period, followed by classification using defined grading systems to evaluate ocular lesions [73].

Ensuring biocompatibility in organic bioelectronics requires a comprehensive approach that considers both cellular responses and whole-system interactions, guided by established regulatory frameworks to minimize adverse effects and maintain reliable experimental conditions between the organic materials and the biological system being studied.

## 3. Organic Materials for Biological Applications

### 3.1. Organic Materials

In bioelectronics, organic materials are widely used due to their biocompatibility, flexibility, and tunable electronic properties. Specific materials are chosen depending on the biological application. These materials can be broadly categorized based on their functions and structures. The main types of organic materials used in bioelectronic applications include the following.

Natural biopolymers: These are naturally derived materials that are structurally modified to be conducting. They ensure excellent biocompatibility or even biodegradability, with natural abundance and sustainability. They include polysaccharides (e.g., cellulose, chitosan, alginate) and protein-based polymers (e.g., silk, gelatin) [74].

Synthetic biopolymers: These are engineered materials that are synthetized chemically or biologically and tailored to specific applications where natural biopolymers may not suffice due to limitations in stability, scalability, or functionality. They include polyurethane, poly(ε-caprolactone) (PCL), and polylactide (PLA) [75].

Organic carbon-based materials: Carbon-based materials are used for their excellent physical and chemical properties, including enhanced thermal, electrical, and optical performance. Carbon nanomaterials include graphene, carbon nanotubes, carbon dots, pentacene, and fullerene C60 [76].

Conducting polymers: These are organic polymers having a backbone consisting of alternating single and double covalent bonds. They conduct electricity and are commonly used for their ability to interact with biological tissues. Typical CPs include polyacetylene (PA), polyaniline (PANI), polypyrrole (PPy), polythiophene (PTH), poly(para-phenylene) (PPP), poly(phenylenevinylene) (PPV), and polyfuran (PF) [6].

### 3.2. Conducting Polymers

Among all organic materials, CPs possess a remarkable ability to combine electrical and optical properties similar to those of metals and the mechanical properties of conventional polymers. This unique combination makes them highly versatile materials in a wide range of applications and positions them at the forefront of cutting-edge research.

**Figure 3:**
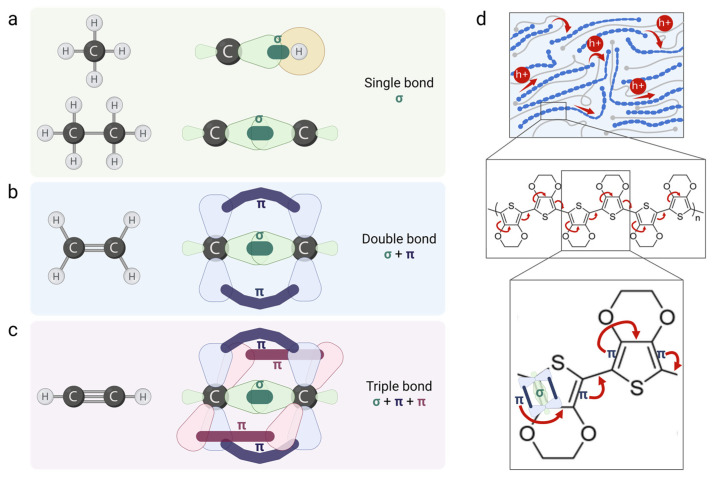
Molecular orbitals and conduction mechanisms in conducting polymers. The location of an electron pair around two or more nuclei and the resulting (**a**) single, (**b**) double, and (**c**) triple bonds pairing the electrons through σ or π covalent bonds. (**d**) The π bond creates a system of delocalized electrons that can move along the CP backbone (i.e., band-like conduction) or hop between localized states within a lattice (i.e., hopping-like conduction).

CPs are polymers with conjugated carbon chain structures, based on a backbone of alternating single and covalent double bonds. Covalent bonds are chemical bonds that involve the sharing of a pair of electrons between atoms. The location and wave-like behavior of the electrons in a molecule are represented by the molecular orbitals (Figure 3a–c). A single covalent bond contains a localized σ bond (i.e., nuclei are connected via the head-on overlap of atomic orbitals), and a double bond has both σ and a weaker π bond (i.e., nuclei are connected via the side-to-side overlap of atomic orbitals) that create a system of delocalized π electrons. The delocalized system and the displacement of the conjugated π bonds along the backbone enable the movement of electrons (i.e., conductivity): the double bond between the first and second carbons includes a weak π bond that will be transferred to the second and third carbons, triggering the transfer of the π bond between the third and fourth carbons and the next pair [6] (Figure 3d).

This mechanism resembles temperature-independent transport and is commonly associated with band-like conduction. In addition, hopping-like conduction is a temperature-induced phonon-assisted mechanism involving the hopping of charge carriers across (i) two adjacent chains when the chains are closely packed or significantly overlap or, alternatively, (ii) two distant parts of the same polymer’s chain when the chains are isolated or coiled [77].

To achieve the high electrical conductivity characteristic of CPs, electrons are added or withdrawn from the polymer backbone through a process called doping. Doping is a strategy used to tune the charge density, and thus conductivity, by elevating the concentration of charge carriers and raising the Fermi level, grading the thermodynamic work required to add an electron to the material. The introduction of charge carriers causes localized lattice distortions, trapping the carriers and forming polarons or bipolarons (i.e., two charge carriers bound together due to strong lattice deformation).

P-type (i.e., positive-type) and n-type (i.e., negative-type) doping use redox reactions to introduce both positive and negative polarons or bipolarons in the polymer structure. In p-type doping, the polymer is oxidized by removing electrons from its structure, creating positively charged regions along the polymer (i.e., holes). In n-type doping, the polymer is reduced by adding extra electrons to its structure, introducing negatively charged regions on the polymer backbone. The conductivity changes radically depending on the doping material, and significant efforts are made to determine the doping process that will result in superior polymer development (e.g., increasing the carrier concentration while maintaining high charge carrier mobility [78]).

CPs are synthetized using different approaches (e.g., chemical, electrochemical, enzymatic, etc.), all having their own advantages, which are chosen according to the final application of the polymer: surface coating, film or powder generation, micro-patterning, biodegradability, etc. [79]. The standard synthetization techniques that are frequently used include the following.

1. Oxidative polymerization: chemical synthesis where monomers are oxidized by an oxidant to generate reactive intermediates that polymerize to form the polymer.
2. Oxidative electrochemical polymerization: electrochemical synthesis where monomers undergo electrochemical oxidation on an electrode surface under an applied electrical potential.
3. Vapor-phase polymerization: a monomer in the vapor phase reacts only with the oxidant deposited on a substrate to form the polymer.
4. Plasma polymerization: gas-phase monomers are introduced into a plasma environment, leading to the formation of a polymer film on a substrate. The plasma (containing energetic species like ions or electrons) creates free radicals on the surface of the polymer that initiate polymerization.
5. Solid-state polymerization: monomers are exposed to heat until the end groups mobilize enough to initiate polymerization in the absence of oxygen or water.
6. Enzymatic polymerization: enzymes catalyze the in vitro polymerization of monomers via non-biosynthetic pathways.

### 3.3. Types of Conducting Polymers

The unique conducting properties of CPs arise from their intrinsic band- and hopping-like charge transport mechanisms, as well as their tunable doping strategies and diverse synthesis approaches. These have led to the development of a wide range of CPs, each tailored to specific applications.

**Figure 4:**
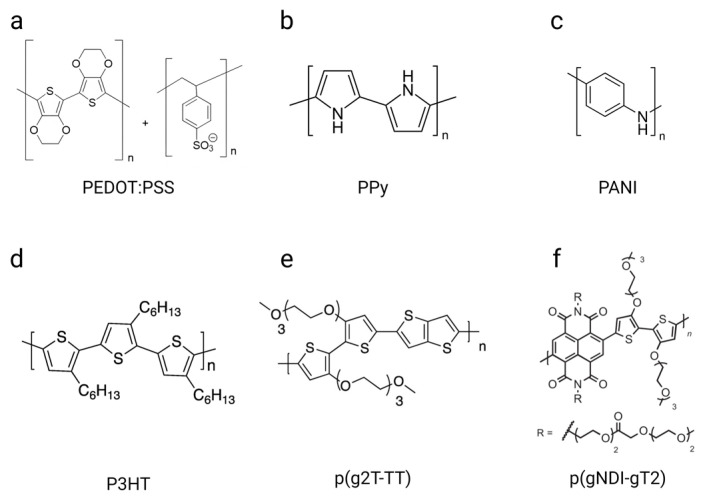
Chemical structures of conducting polymers. (**a**) Poly(3,4-ethylenedioxythiophene):poly(styrene sulfonate) (PEDOT:PSS). (**b**) Polypyrrole (PPy). (**c**) Polyaniline (PANI). (**d**) Poly(3-hexylthiophene-2,5-diyl) (P3HT). (**e**) Poly(2-(3,3′-bis(2-(2-(2-methoxyethoxy)-ethoxy)ethoxy)-[2,2′-bithiophen]-5-yl)thieno[3,2-b] thiophene) (p(g2T-TT)). (**f**) Poly((ethoxy)ethyl2-(2-(2-methoxy ethoxy)ethoxy)acetate)-naphthalene-1,4,5,8 tetracarboxylic-diimide-co-3,3ʹ-bis(2-(2-(2-methoxy ethoxy)ethoxy)ethoxy)-(bithiophene)) (p(gNDI-T2)).

Polyaniline (PANI) and polypyrrole (PPy) (Figure 4b,c) were the very first polymers reported to exhibit semiconductive properties. PANI was the first reported synthetic organic polymer and was publicly described in 1862 [80], while PPy was introduced in 1968. Both polymers have been of great interest due to their ease of synthesis by chemical or electrochemical oxidative polymerization and potential for technological innovation. Since the 1980s, research has grown steadily; today, these two polymers are benchmarks in the field of bioelectronics.

Following the development of PANI and PPy, a plethora of CPs have been developed extensively. These now include poly(phenylenevinylene) (PPV), polyacetylene (PA), poly(para-phenylene) (PPP), polyfuran (PF), and polythiophenes (PTH). PTH and its derivatives are the most investigated and used CPs today due to their strong optical properties, ease of processability, and high stability. In particular, poly(3,4-ethylenedioxythiophene):poly(styrene sulfonate) (PEDOT:PSS) (Figure 4a) is a prototypical system for organic bioelectronics and has been extensively used for applications including organic electrodes, solar cells, light-emitting diodes, wearables, and implantable devices, among others [81,82,83].

First synthetized and commercialized in 1988, PEDOT:PSS is composed of positively charged and p-doped PEDOT and negatively charged and water-soluble poly(styrene sulfonic acid) (PSS). PEDOT arises from the polymerization of EDOT, a small organic molecule and monomer. The π-conjugated system of the thiophene rings is oxidized, leading to the formation of positive charges on the polymer backbone. However, PEDOT is difficult to synthetize due to its insolubility and infusibility in many solvents. Therefore, doping it with PSS, a water-soluble counterion possessing negatively charged ester sulfate functional groups, enables stabilization, promotes good ionic mobility, and provides a matrix for PEDOT to form an aqueous dispersion while maintaining its properties. Three major interactions exist in PEDOT:PSS: (i) electrostatic interactions between the PEDOT cation and PSS anion, (ii) π–π stacking between PEDOT’s adjacent chains, and (iii) interchain entanglements between long PSS chains [84]. These interactions provide PEDOT:PSS with key features for biological applications, including superior stability [85], high conductivity [86,87], good transparency, flexibility [81], water solubility, and biocompatibility [88,89].

Another widely used and studied derivative of PTH is poly(3-hexylthiophene-2,5-diyl) (P3HT) (Figure 4d). P3HT possess excellent optoelectronic properties, processability, and stability. Its ability to efficiently produce electricity by absorbing visible light makes it an ideal material for solar cell applications but also for wireless electrical supplies for bioelectronic devices, as well as the electrical stimulation/photoactivation of biological samples using optogenetics [90,91].

New types of CPs have recently gained attention in the field of organic electronics, including poly(2-(3,3′-bis(2-(2-(2-methoxyethoxy)-ethoxy)ethoxy)-[2,2′-bithiophen]-5-yl)thieno[3,2-b] thiophene) (p(g2T-TT)) (Figure 4e) and poly((ethoxy)ethyl2-(2-(2-methoxy ethoxy)ethoxy)acetate)-naphthalene-1,4,5,8 tetracarboxylic-diimide-co-3,3ʹ-bis(2-(2-(2-methoxy ethoxy)ethoxy)ethoxy)-(bithiophene)) (p(gNDI-gT2)) (Figure 4f). They were both first synthetized in 2016 for OECT applications [92,93]. p(g2T-TT) is a polythiophene copolymer with grafted glycol side chains, achieving high currents at the submillisecond time scale, stability in continuous operation in aqueous solutions, and superior OECT transductance compared to PEDOT:PSS-based OECTs of the same geometry. p(gNDI-gT2) is also a polythiophene polymer but is based on an alternative naphthalene-1,4,5,8-tetracarboxylic diimide (NDI) and bithiophene (T2) polymer backbone. It has been developed as the first n-type OECT that presents ambipolar properties, meaning that it can both support holes and electron transport along its backbone. The development of such n-type CPs is crucial to create electronic circuits, as CMOS technology requires both a p-type and n-type metal–oxide semiconductor.

These CPs exhibit a combination of conductivity, mechanical properties, and biocompatibility, making them highly suitable for several biomedical applications (Table 1 and Table S1). Ongoing advancements in the development of CPs continue to enhance their performance and explore new avenues to find composites, derivatives, or materials that are more efficient for specific applications. Specifically, a great deal of attention is paid to ensuring that these materials meet critical requirements, including (i) biocompatibility to avoid toxicity when in contact with biological samples, (ii) stability in electrolytes or culture media, (iii) mixed ionic and electronic conduction, and (iv) mechanical properties matching those of tissues [9].

### 3.4. Advantages of Organic Materials

When integrated with biological systems, electronic devices using organic materials offer numerous advantages, including mechanical compatibility, biocompatibility, mixed ionic and electronic conduction, ease of processing, flexibility, a light weight, and optical transparency [64,69,114,115].

**Figure 5:**
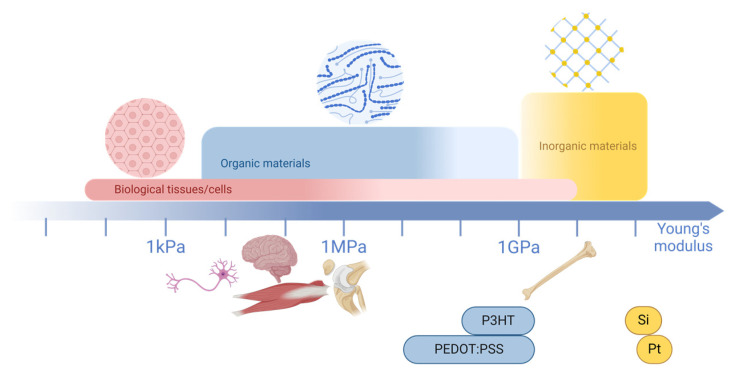
Comparison of Young’s modulus ranges of biological tissues or cells, organic materials, and inorganic materials, with different examples represented below the scale. It is shown that the mechanical properties of biological tissues are more closely reflected in organic materials than inorganic materials [9,69,107,116,117,118,119,120,121,122,123,124,125,126,127].

Organic materials are carbon-containing molecules where the different atoms are covalently bonded to each other. They then form (macro-)molecular blocks, which are held together by weak van der Waals interactions, and, in the case of doped materials, electrostatic interactions are also involved. In contrast, inorganic materials such as gold, platinum, and silicon do not contain carbon atoms and are held together by a network of covalent bonds, where each atom shares valence electrons with four neighbors. This is the case for silicon, which is an inorganic material that is traditionally used in (bio)electronic devices. The soft properties of organic materials, in contrast to the rigid proprieties of inorganic ones, is observed as a result of van der Waals interactions [9,115]. Thus, the mechanical properties of organic bioelectronic devices offer significant advantages over inorganic ones. For instance, when comparing the Young’s modulus of a biological tissue, such as the brain, to that of silicon, the difference is substantial. Inorganic materials are approximately six orders of magnitude stiffer than brain tissue. A comparison of the different ranges of the Young’s modulus can be found in Figure 5, where it is shown that organic materials’ mechanical properties are closer to those of biological systems than inorganic ones [69,116]. Specifically, for CPs, their Young’s moduli are compared with those of other types of materials in Table 2.

Furthermore, organic materials (and specifically CPs) offer flexibility [128], bendability [129], and stretchability [61,130] when interfacing with biology (Table 2). For instance, azobenzene polymer films recently showed the capability to enwrap neuronal structures, a property called conformability, when triggered by light [131]. The above mechanical properties can be highly beneficial in integration with various biological tissues, providing a more precise platform for guidance [131]. Due to their flexibility and ability to adopt multiple forms, organic bioelectronic materials provide superior cell–electrode coupling. This enhanced coupling facilitates the more efficient transduction of biological events. For example, PEDOT:PSS 3D scaffolds may serve a dual purpose by hosting cells as a scaffold and recording their activity within their environment [9,132,133]. These mechanical properties of organic materials show that they are well suited to biological systems.

**Table 2:** Comparison of different characteristics of conducting polymers with other materials such as metals, graphene, and MXenes in the context of bioelectronic devices.

		CPs	Metals	Graphene	MXenes
Sensitivity	Electrical conductivity	10^−10^ to 4380 S/cm [86,87,102]	10^5^ to 6.8 × 10^5^ S/cm [134]	106 S/cm [135]	100 to 24,000 S/cm [136]
	Electron mobility	10^−6^ to 10^−4^ cm^2^/Vs [137,138,139]	5460 to 37,590 cm^2^/Vs [140]	200 to 2 × 10^5^ cm^2^/Vs [135,141]	10^6^ cm^2^/Vs [142]
Mechanical properties	Young’s modulus	2 MPa to 5 GPa [85,99,100]	72 GPa to 410 GPa [143]	1 TPa [135,144]	0.33 ± 0.03 TPa [145]
	Flexibility	+ +	− −	−/+	−/+
Biocompatibility		+ + +	+ +	+	+ +
Cost		USD 0.30 to 10 per gram [146]	USD 30 to 96 per gram (subject to fluctuations due to market dynamics) [147,148,149]	USD 1.12 per gram [150]	USD 20.33 per gram [151]

The symbols − −, −/+, +, + +, + + + indicating levels of intensity, from poor to very good.

Another important property of organic materials is their mode of conduction. Particularly in CPs, charge carriers include both ions and electrons (or holes) (Table 2). In contrast, silicon, from which most electronic devices are fabricated, conducts only electrons (or holes). Since biological systems primarily use ions for communication, there is a communication gap between biology and electronics [69]. The goal of bioelectronics is to monitor and/or stimulate biological systems, and mixed ionic/electronic conductivity is essential for improved biotic/abiotic communication. CPs support both ionic and electronic conduction. Electronic conduction requires the movement of electrons or holes toward the material, which can also occur in inorganic ones, as this occurs with semiconductors or metals. This includes mechanisms such as hopping or band-like transport (see Section 3.2). Ionic conduction is additionally identified, which includes the diffusion of charged ions [152]. Electronic charge carriers like holes or electrons must be stabilized by counterions in CPs. For example, in PEDOT:PSS, the positively charged holes on the PEDOT backbone are stabilized by the doping anionic counterion PSS. This process not only maintains charge neutrality but also allows for efficient transport. As a result, PEDOT:PSS, like other CPs, becomes a highly conducting and stable material, ideal for various applications. When a potential is applied, it modulates the coupling between ionic and electronic charges, driving key processes like charge storage, signal transduction, and conductivity control. For example, in PEDOT:PSS-based OECTs, a positive gate bias drives cations from the electrolyte into the PEDOT:PSS channel, where they neutralize holes in PEDOT by compensating for negatively charged PSS sites, thereby decreasing the channel’s electrical conductivity [153,154,155]. The positive applied potential drives cations from the electrolyte into the channel, where they neutralize the negatively charged PSS and remove the holes in PEDOT. This reduces the channel’s electrical conductivity, which is detected by the device. Changes in the ionic concentration in the electrolyte are then converted into changes in electrical conductivity. The response of the system depends on the channel’s volume, where larger volumes lead to higher transconductance, as they can store more charge. This mixed ionic–electronic conduction improves signal transduction from biology to electronics.

In addition, organic materials in bioelectronics offer remarkable advantages in bioelectronic applications due to their chemical tunability, ease of processing, optical transparency, and biocompatibility (Table 2). Their tunable chemical composition allows the customization of their mechanical and electrical properties, as demonstrated by blending additives with CP dispersions [113]. They also show ease of functionalization, which enables innovative applications [128]. Organics are easily processed through low-cost techniques like printing and solution deposition [128,156], offering biocompatible, flexible devices [157]. Moreover, their optical transparency facilitates multimodal transduction and expands their applicability in wearable electronics [9,158]. Organic materials have also shown promising results in biocompatibility tests by minimizing cytotoxicity and supporting long-term cell viability [69].

In summary, organic materials, particularly CPs, offer advantages for bioelectronics, including mechanical properties that match those of biological systems, mixed ionic–electronic conduction, and biocompatibility, providing them with excellent features to interface with biological systems, as compared to other types of materials, such as metals, graphene, and MXenes. A comparison of their characteristics is depicted in Table 2. However, assessing representative values for stability, specificity, and scalability is challenging due to variations influenced by multiple factors, such as the film thickness, processing method, and bulk material.

## 4. Organic Bioelectronic Devices and Their Fabrication

The advantages of organic materials have driven their integration into bioelectronic devices, where they serve as essential components for signal transduction between biological and electronic systems.

**Figure 6:**
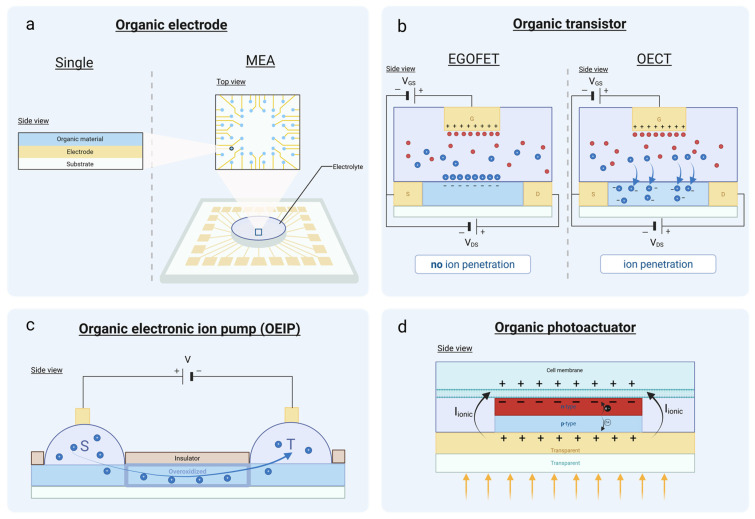
Examples of the different types of organic bioelectronic devices. (**a**) An organic electrode, where a single and a MEA are represented. The single electrode is composed of a substrate, onto which an electrode is coated with an organic material. (**b**) An organic transistor, where an EGOFET and an OECT are represented. The latter are composed of three metal electrodes: the gate (G), the source (S), and the drain (D). An initial positive bias is applied between G and S in the two devices. In the EGOFET, two electrical double layers (EDLs) are then formed (one at the interface between the organic material and the electrolyte and one at the interface between G and the electrolyte) and no penetration of ions is found. In the OECT, after the positive bias, the positive ions found in the electrolyte penetrate into the organic material, creating volumetric capacitance. (**c**) An OEIP, where a positive bias is applied between the source and the target. The overoxidized layer of organic material then permits the selective flow of positive ions from the source to the target. (**d**) An organic photoactuator is represented under a cell membrane, where the n-type and p-type parts of the organic material lead to the separation of light (yellow arrow above) into an electron and a hole. An EDL is then created between the cell membrane and the n-type material following the movement of electrons at the interface, and an ionic current is also created to neutralize the holes, transferred into the metal electrode.

A bioelectronic device is a transducer that conveys signals from biology to electronics and vice versa [115]. For example, when a biological phenomenon takes place, a biosensor can detect this event and translate it into useful information in the form of electrical signals. This is how we can monitor biologically relevant processes and sense abnormalities in the operation of biological systems. Bioelectronic devices can also act as actuators and trigger events in the biological world. In this way, we can intervene in biological systems and use currents, or any other physical means, to change cell behavior or even to treat the symptoms of diseases in a mode of operation that is leveraged in the field of bioelectronic medicine [159]. Organic materials have recently gained increased attention among the scientific community for the fabrication of state-of-the-art bioelectronic devices due to their unique characteristics. Next, we will present the basic organic devices (Figure 6) that are used in bioelectronics as biosensors and actuators, and we will discuss the basic principles of their operation, their main applications, and their fabrication methods. 

### 4.1. Organic Electrodes

At biological interfaces, electrodes are the simplest forms of transducers used to convey messages from the domain of electronics to the domain of biology and vice versa. These electrodes are typically made from inorganic materials (e.g., gold, steel, platinum, or silicon) and they allow the interrogation of biological tissues by enabling both the recording and stimulation of biological activity [9,160].

This interaction can be manifested in the recording of APs and the local field potential of neurons. Electrodes can record APs by detecting changes in electrical activity or trigger it by applying a current or voltage, thereby inducing a change in the electrical state of the cells.

Electrodes can be characterized by their impedance (Z). Impedance quantifies the opposition that a system presents to the flow of an alternating current (AC). It is a combination of resistance (R) and reactance (X), where reactance includes contributions from capacitance (C) and inductance (L). Impedance is measured in ohms (Ω) and can be expressed mathematically as [161]

$$\left|Z\right|=\sqrt{R^{2}+X^{2}}$$

where X equals either $X_{C}$ or $X_{L}$ (as represented below) or a combination of both, depending on the type of circuit [161]:

$$X_{C}=\frac{1}{2\pi fC}\;and\;X_{L}=2\pi fL$$

It is important for electrodes to present low impedance to monitor the biological activity of tissues and cells effectively.

Electrodes are valuable tools in obtaining information about biological tissues and cells. For example, they are used in techniques like intracellular recording (for example, in patch clamps) and extracellular recording (for example, in intracranial electrodes) to measure cellular activity [162,163]. However, in order to obtain a spatial recording of a biological tissue, there is a need to multiply the number of electrodes in a single device, meaning that the size of the electrodes should be reduced. Additionally, the need to record the activity of individual cells dictates the miniaturization of electrodes to the micrometric scale in order to match the cell size [164].

MEA devices have been developed by integrating multiple electrodes into a single system to transduce electrical signals into biological responses and vice versa, while capturing spatial information (Figure 6a). These millimeter-scale devices consist of electrodes (10 to 100 µm [165,166]) patterned on a substrate with an insulation layer (defining the size of the electrodes) [167,168]. Depending on the specific application, both the substrate and insulation materials are selected accordingly. Additionally, the size, material, and geometry of the electrodes are adjusted to meet the requirements of the application [169]. This approach resolves the issue of spatial resolution, allowing cellular and tissue activity to be quantified at multiple locations within biological tissues and cells [164].

However, reducing the electrode’s size imposes a penalty on their impedance value, because the impedance scales inversely with the area of the electrode, as shown already [170,171]. Reducing the geometric area of electrodes to integrate them into the same device increases the impedance of the recording site [172]. To overcome these hurdles, specific materials are used, namely organic materials, and we will focus here on CPs. By coating electrodes with CPs, the impedance is significantly reduced due to their mixed ion–electronic conductivity, which results in a capacitance increase and therefore, an impedance decrease in the biotic–abiotic ensemble. Lower impedance is linked to a higher SNR, which improves the electrodes’ sensitivity to detect smaller ionic signals, improving their performance in biological environments [9,173].

CPs coated on MEAs improve the capacity to record and stimulate biological activity, with improved precision and signal quality. Specifically, when recording neural activity, the process can be separated into several steps. A physiological impulse triggers the movement of ions, such as sodium and potassium, across the cell membrane. This ion flow causes a localized shift in ionic concentrations, which then propagates through the electrolyte and reaches the electrode, where the change is detected. Regarding the stimulation of neural activity, the reverse process occurs when the metallic electrodes initiate ionic motion in the electrolyte. This is achieved through controlled charge injection methods, such as regulating the applied electrical current or voltage, which generate an electric field and induce a physiological impulse [169].

For example, an improvement in recording quality with coated CPs was reported with higher SNRs in multiple PPy- or PEDOT:PSS-coated electrodes within a MEA, as compared to uncoated electrodes [160]. This was also proven for a neural recording electrode coated with PEDOT:PSS compared with uncoated controls, satisfying the need to reduce the size of the electrode while ensuring a good-quality signal [174].

Organic electrodes have the potential to improve bioelectronic interfaces by reducing impedance noise and improving signal transduction, making them highly effective for the recording and stimulation of biological applications. Alongside these passive components, organic transistors introduce additional functionalities, such as signal amplification, expanding the possibilities for bioelectronic devices.

### 4.2. Organic Transistors

The goal of bioelectronic devices is to record and stimulate biological signals through electrical interfaces while achieving the sensitivity needed to detect even the smallest signals. To enhance this sensitivity, optimization is crucial. A device initially used in computing applications, the transistor, has demonstrated remarkable potential in the biological field [175]. A transistor is a three-terminal device that consists of a semiconductor placed between two metal electrodes: the source and the drain. The semiconductor forms an active channel where charge carriers flow. Additionally, a critical third electrode, the gate, allows for the precise modulation of the current through the channel, enabling the transistor to control and amplify signals effectively [9,176]. There are then two characteristic voltages involved in the transistor’s operation: one that is applied between the source and the gate (V_GS_) and one that is applied between the source and the drain (V_DS_).

While MEAs can record and stimulate biological signals, they cannot amplify them. Transistors, in contrast, not only allow for recording and stimulation but also offer the advantage of signal amplification [177,178]. This capability makes them highly sensitive to weak signals, significantly enhancing their performance in biological applications [179].

Among the various types of organic transistors, electrolyte-gated organic transistors (EGOTs) are particularly interesting for bio-applications because they integrate electrolytes, which are abundant in biological systems, into their operating principles. Within EGOTs, two main subtypes exist, EGOFETs and OECTs, depending on the ionic permeability of the channel. EGOFETs refer to the mode of operation in which ions from the electrolyte do not penetrate the bulk of the channel, while OECTs involve the drifting of ions from the electrolyte into the bulk of the channel [176,180].

The structural difference between EGOFETs and OECTs lies in their semiconductor material (Figure 6b) [181].

#### 4.2.1. EGOFET

EGOFETs are organic transistors that operate using an electric field to modulate the charge carriers in an electrolyte, which in turn influences charge transport in the semiconductor channel, enabling them to regulate the charge dynamics in the studied system. These devices consist of three primary electrodes typically found in transistors: the gate, source, and drain. Their channel is composed of an ion-impermeable organic semiconductor material (e.g., P3HT) [182]. When a voltage is applied at the gate, electrical double layers (EDL) are formed at two critical interfaces: one between the gate and the electrolyte and the other between the electrolyte and the channel. An EDL consists of two surfaces carrying opposite charges, positive on one surface and negative on the other, which together act as a capacitor-like structure [176,180].

The modulation of the charges induced by the EDLs is referred to as the field effect, since the formed electric field controls the current flow between the source and the drain [183]. Historically, these devices were first constructed using oxides as the dielectric material separating the channel from the organic channel, rendering the latter impermeable to ions. Although such device architectures enabled fast operation due to surface-limited gating, they require the application of high gate voltages to move charges effectively. However, in an aqueous environment, voltages exceeding 1 V present a significant challenge, as they can lead to water electrolysis. This phenomenon disrupts biological systems and is particularly problematic when working with solutions or tissues that rely on a stable aqueous environment [184].

Consequently, there have been research efforts dedicated to reducing the voltage requirements of these devices to make them compatible with biological environments, while maintaining their operational efficiency [181].

EGOFETs can be fabricated using photolithography and lift-off techniques and have a channel length in the micrometer scale [185]. They demonstrate high reproducibility in sensing biomolecules and modulating cell activity, with robust functionalization strategies enhancing their sensitivity [181,185]. Additionally, they exhibit up to 10 days of stability under biological conditions such as cardiomyocyte monitoring, making them effective for applications like electrophysiological testing [181].

An exemplary EGOFET biosensor was developed using pentacene as the organic semiconductor channel, with a recognition unit added to its surface. Designed to detect tumor necrosis factor α (TNFα), a pro-inflammatory cytokine, the biosensor demonstrated a low detection limit and high sensitivity. This type of device represents a promising step toward early diagnostics in healthcare [185].

EGOFETs represent a significant advancement in organic transistors, offering a promising approach to interfacing with biological systems by modulating charge dynamics through an electric field, while addressing challenges related to voltage requirements in biological environments.

#### 4.2.2. OECT

OECTs are similar to EGOFETs, but, instead of using an electric field, they modulate charge carriers through ion penetration into the semiconductor, leading to a measurable change in its conductivity. While OECTs share a similar structure with EGOFETs in terms of the gate, source, and drain electrodes, the key difference lies in the channel material (with a length also in the micrometer scale), which is an ion-permeable organic material. This modification enables OECTs to operate at significantly lower voltages (0 V–0.6 V) [9], which is particularly advantageous for biological systems that function in aqueous environments.

The ion permeability of the channel material in OECTs allows for efficient ionic and electronic coupling, leading to a notable property: high transconductance (gₘ). Transconductance (gₘ) measures the amplification capability of the device. It indicates the resulting current at the drain (V_DS_) that is recorded when a gate voltage (V_GS_) is applied. In simpler terms, when using a device characterized by high transconductance, even when a low voltage is applied at the gate, the resulting current recorded at the drain is considerable, demonstrating the device’s ability to amplify small signals effectively [9,186].

OECTs can operate in two distinct opposite modes, determined by the doping state of the organic channel material. In depletion mode, which occurs when the channel is already conducting (as in the case of PEDOT:PSS), applying a gate voltage depletes the charge carriers, transitioning the device from an ON state to an OFF state. In contrast, in accumulation mode, where the channel material is initially non-conducting (such as p(g2T-TT)), applying a gate voltage causes the charge carriers to accumulate, switching the device from the OFF state to ON [9,176,178,187].

The architecture of OECT devices can be designed in various configurations to optimize the fabrication simplicity. It has also been shown that adjusting the device structure, such as modifying the electrode placement or dimensions, can significantly affect the quality of the recorded signal [176,186]. Vertical OECT geometries are becoming popular due to their high transconductance and the small footprint that they offer [188,189,190].

OECTs offer tunable dimensions that can be used to influence their performance, with larger devices excelling at low frequencies for barrier tissues and smaller ones providing faster responses for non-barrier tissues [178,181]. Their fabrication is highly versatile, utilizing techniques such as lithography, printing, and spin coating to create flexible, cytocompatible, and high-transconductance devices suitable for bioelectronic applications [37,178,186]. OECTs also demonstrate strong reproducibility and long-term stability in biological environments, with the recorded functionality lasting several weeks in aqueous conditions and up to 42 days in cardiomyocyte monitoring [178,181].

The ability of OECTs to combine low-voltage operation and high transconductance (and compatibility with aqueous systems) makes them extremely appealing in bioelectronic applications However, it is worth noting that EGOFETs, with their surface-limited gating mechanism, offer faster response times compared to OECTs, showing the complementary advantages of both technologies depending on the application.

An OECT device using PEDOT:PSS as the active layer was developed for an in vivo model to monitor cancer and fibroblast cell activity. The device detected interactions between the cells and the CP under varying conditions, such as the addition of retinoic acid, providing insights into cell behavior. It demonstrated good stability and biocompatibility in the cell medium [191].

OECTs offer an adaptable and highly effective solution for bioelectronic applications, thanks to their ion-permeable channel, which enables efficient ionic and electronic coupling. Their ability to operate at low voltages, their high transconductance, and their compatibility with aqueous environments make them particularly well suited for interfacing with biological systems.

### 4.3. OEIPs

OEIPs are devices capable of selectively transporting ions from one location of a biological system to another, making them promising tools for targeted drug delivery applications [4] (Figure 6c).

OEIPs consist of two electrolytes, each connected to an electrode. The first electrolyte, known as the source, is where the ions (or drugs, in specific applications) originate, while the second electrolyte, referred to as the target, is where the ions are delivered, typically in contact with biological tissues or cells [187]. These two electrolytes are separated by a membrane composed of an overoxidized organic material, which plays a key role in enabling ion selectivity. Overoxidation modifies the material to selectively allow ions to pass through while blocking electrons and holes [192]. When a potential is applied between the two electrodes, ions from the source electrolyte migrate through the overoxidized membrane to the target electrolyte. The flow of ions is controlled by the magnitude of the applied current. Importantly, the charge of the membrane determines which ions can pass through it, where only ions with the opposite charge to the membrane’s will be transported.

The construction of these devices relies on photolithographic patterning and electrodeposition [193], where the dimensions of the microchannel and arrays (micrometer scale) and the length (millimeter to centimeter scale) are usually tailored to the biological system being studied, such as eukaryotic or neuronal cells [194,195]. The reproducibility can be enhanced through Pd-based processes, ensuring consistent drug delivery [193], while their stability was demonstrated without adverse effects on rats’ motor function [195].

In a study, they successfully demonstrated the ability to retain spatial information by creating an array of OEIPs for targeted neurotransmitter delivery both in vitro and in vivo [195,196].

The key advantages of these devices lie in their ability to selectively transfer specific ions, ensuring the specific delivery of components. Additionally, they are less invasive compared to other methods since they do not rely on liquid flow for operation [4,192].

OEIPs stand out for their ability to selectively transport ions between electrolytes, making them valuable tools for targeted drug delivery and other bioelectronic applications. Their ion-selective membranes, controlled by the applied current, allow for precise delivery, while their microchannel and array configurations can be tailored to various biological systems.

### 4.4. Organic Photoactuators

Organic photoactuators are devices that convert light into electrical signals to trigger biological activity (Figure 6d). They consist of a micrometer- to centimeter-scale transparent substrate, typically glass, coated with a light-sensitive organic material that is sensitive to light [197], and they typically consist of two layers: an n-type (electron donor) and a p-type (electron acceptor). When light is absorbed by the device’s active layers, pairs of electrons and holes are created. The electrons migrate to the n-type layer, interfacing with the electrolyte and creating an EDL, while holes are inserted into the metal electrode. The holes are neutralized by the ionic current of the electrolyte.

There are two main types of photoactuators, based on their operating mechanisms: photothermal actuators and photovoltaic actuators [9,192]. The photothermal mechanism occurs when the induced light is converted into heat, warming the device. The heat then spreads through the biological system, which is in direct contact with the device, creating a temperature gradient. This gradient affects the biological activity by altering the cell membrane’s capacitive and resistive properties, which in turn influence ion transport across cell membranes, potentially leading to biological triggering. Photovoltaic actuators can be categorized based on two distinct mechanisms: photocapacitive, where light triggers the formation of an EDL between the electrolyte and the semiconductor, and photofaradaic, where light induces redox reactions to modulate biological activity [192,198,199].

In the context of neuronal stimulation for retinal improvement, a device using a photoactuator based on the CP PEDOT:PSS was developed. The simplicity of the device structure is a significant advantage, although stability issues and the need to achieve a sufficiently high current still need to be addressed [192]. Organic photoactuators are devices that convert light into electrical signals to stimulate biological activity, offering distinct mechanisms of operation. Whether through photothermal or photovoltaic effects, they provide a promising approach to modulating biological systems in various applications.

In summary, organic bioelectronic devices, including organic electrodes, organic transistors, OEIPs, and organic photoactuators, are unique in their ability to convert biological signals into electrical ones, each offering distinct advantages over traditional inorganic alternatives. The integration of organic materials is thus a crucial step in the development of these devices, highlighting the importance of advancing the (micro)fabrication of organic bioelectronics.

## 5. Organic Material Patterning and Biofunctionalization Methods

While these devices are fabricated using standard microfabrication techniques, including photolithography, metal deposition (via sputtering or thermal evaporation), and etching, this review primarily focuses on the patterning and biofunctionalization of organic materials.

### 5.1. Organic Material Patterning

**Figure 7:**
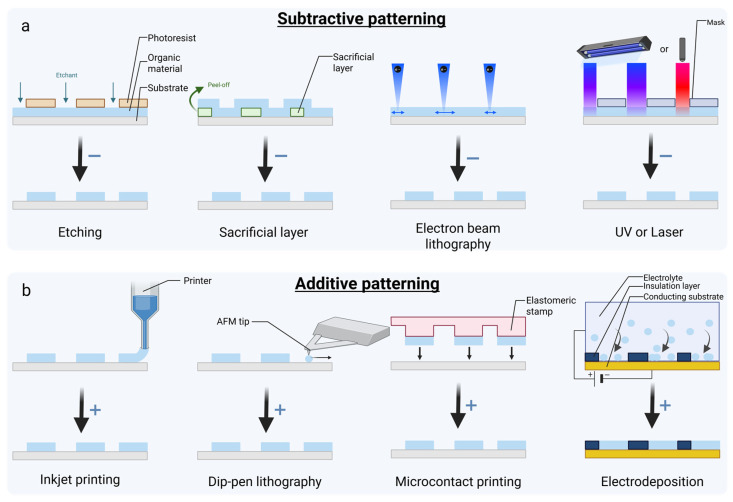
Illustration of two different types of patterning methods: subtractive and additive patterning. (**a**) Subtractive patterning, where the desired layer is achieved by first depositing the organic material uniformly across the substrate’s surface and then removing the undesired layer using various techniques. These include etching, where the undesired material is removed using a photoresist and an etchant; lift-off, where a sacrificial layer is first applied, followed by the deposition of the organic material and finally by peeling off the sacrificial layer to retain the desired layers; and electron-beam lithography, UV, or laser patterning, where the undesired material is removed by applying an electron beam, UV light, or a laser, respectively. (**b**) Additive patterning, where the desired layer is directly applied onto the substrate using techniques such as inkjet printing, where a printer deposits the material; dip-pen lithography, which uses an AFM probe; microcontact printing, where an elastomeric stamp coated with the organic material at its reliefs deposits the material; and electrodeposition, where the organic material is deposited on a conductive substrate after the application of a current [114,165,187,200].

The fabrication of organic bioelectronic devices involves several steps to deposit organic materials onto substrates. The aim is to create a thin, uniform layer with the desired pattern or structure. There are various methods to achieve this. Some techniques require separate deposition and patterning steps, while others can simultaneously deposit and pattern the material. The former techniques are categorized as subtractive methods because, after layer deposition, extra steps are used to remove the material in order to pattern the polymer layers. The latter ones are called additive methods in this review, because the desired layer is directly deposited on the surface. Below, common methods are explored for deposition, patterning, and combined approaches; these are often cited in the literature and are represented in Figure 7 [114,165,187,200].

For the deposition of organic films, various techniques are used, such as spin coating, electrodeposition, chemical vapor deposition, and spray coating. Spin coating, one of the most widely used methods, involves depositing a material onto a surface and then spinning the surface rapidly. This spreads the material evenly to form a thin film. While spin coating is straightforward and covers large areas of the substrate, it may lack uniformity on non-flat surfaces.

After deposition, patterning methods are used to create customized designs in the organic material, enabling greater flexibility in the device architecture by removing undesired layers. In Figure 7a, different subtractive patterning methods are illustrated, including etching, sacrificial layer, electron-beam lithography, UV, and laser methods. Etching is one of the patterning methods in which a photoresist material covers certain areas, seeking to retain the organic layer. An etchant (liquid or plasma) removes unprotected material, leaving the desired pattern. The photoresist is then removed with other specific solvents, completing the process. Another approach is the sacrificial layer technique, where a pre-patterned layer is deposited before the organic material. After deposition, the sacrificial layer is removed manually or chemically with a solvent, leaving only the organic material layer. Electron-beam lithography offers high-resolution patterning by adding or removing nanoscale features using an electron beam. Finally, UV or laser techniques work by exposing specific areas of the organic material to UV light through masks, altering its properties to resist removal. Unexposed areas are then dissolved with a developer solution [114,165,187,200].

In Figure 7b, different additive patterning methods are represented. These techniques include inkjet printing, dip-pen lithography, microcontact printing, and electrodeposition. In order to pattern the material during deposition, a few different techniques can be employed. Inkjet printing allows the direct deposition of organic materials onto substrates with customized patterns. Dip-pen nanolithography uses an atomic force microscopy (AFM) tip to deposit nanoscale compounds with precision, making it of interest for organic or biological materials. Microcontact printing employs an elastomeric stamp with a relief pattern. The stamp transfers the material to the substrate only at the contact points, offering a simple and efficient patterning method. Finally, electrodeposition is another technique that uses an electric current to deposit the organic material. An insulation layer is pre-deposited on a conductive substrate and placed in an electrolyte, where the applied current induces the deposition of the organic material on the conductive substrate.

Due to the deposition and patterning techniques discussed, the fabrication of organic bioelectronic devices enables precise control over material application and design. These methods are essential in enhancing and tailoring the performance and functionality of bioelectronics.

### 5.2. Biofunctionalization

Biofunctionalization involves the addition of biomolecules or chemical compounds to a material in order to change its properties for biological compatibility. The material’s modification can be performed before or after its deposition and patterning on a substrate [201,202], and it can be achieved through either non-covalent or covalent bonding.

Non-covalent modifications enhance CPs’ properties by integrating biomaterials or dopants through physical interactions. For example, doping PEDOT with carboxyl-functionalized multi-walled carbon nanotubes improved the long-term stability of chronic spike recordings over four months compared to traditional PEDOT:PSS [202]. Another example is the addition of an alginate layer to PEDOT:PSS to improve the biomechanical properties of neuronal tissue and reduce the SNR [203].

Covalent modifications involve the chemical functionalization of monomers, polymers, or dopants through the addition of specific functional groups. This has been highlighted by improved CP biocompatibility through the functionalization of EDOT monomers with an Arg-Gly-Asp (RGD)-like peptide prior to electropolymerization, promoting cell adhesion (monomer functionalization) [202].

Another example is the modification of PPy by introducing an alkylcarboxylic acid before polymerization. After coating the polymer on ITO, the protective group was removed, and CCR4 antibodies were attached, creating a functional sensor for prostate cancer biomarkers while maintaining electrical and mechanical stability (polymer functionalization) [201].

A similar approach was used in dopant modification, where PPy doped with chondroitin sulfate was deposited on gold electrodes, followed by adding collagen to the dopant to create a structure that enhanced cell adhesion and differentiation (dopant functionalization) [204].

In summary, by selecting the appropriate functionalization strategies, researchers can develop tailored sensors for applications ranging from diagnostics to implantable bioelectronics, without compromising the device’s performance or reusability.

## 6. In Vivo Applications

Bioelectronic devices include wearables, which are typically used to monitor physiological processes such as the pulse, sleep, and heart rate, but also implantable devices, which are used for biomolecular quantification and the characterization of cellular activity, as well as in restoring or replacing the functioning of impaired tissues and organs. Due to the integrated connection with the human body in both wearables and implantable devices, biocompatibility is a critical factor to prevent cytotoxic responses. Additionally, stability and durability are essential to ensure long-term functionality.

Surgical implantation, while often necessary, is an invasive procedure and presents various risks. The use of organic materials in bioelectronic devices offers a promising path to reduce these risks due to their mechanical properties. Additionally, their electrical features lead to improved quality of recording. Furthermore, certain organic bioelectronic materials exhibit degradability, paving the way for bioresorbable devices that could reduce the need for additional surgeries or long-term implantation [205].

Here, we present a review of the in vivo bioelectronic devices developed over the past six years using CPs. We classify these devices based on their electrical recording or stimulation functions, as well as their application in implantable or wearable formats for both human and animal models.

CPs hold great promise for neural implantable devices and have been the focus of the majority of in vivo research to date. However, further in vivo validation in animal models is needed to advance these devices toward clinical trials and regulatory approval [192,206]. Through a search on clinicaltrials.gov for “conducting polymers”, only one relevant clinical trial was found (Table S2) (conducted in March 2025). The identified clinical trial compared two rehabilitation interventions following a hip fracture. One intervention targeted exercise-based interventions (active treatment) and the other included a transcutaneous electrical nerve stimulation (TENS) therapy based on a CP wearable device, seeking to determine which intervention served to better restore community ambulation in older adults after hip fractures. This clinical trial has been completed, and the published results state that there was no statistically significant improvement in the ability to walk 300 m or more in 6 min after 16 weeks when the active treatment was compared to the active control TENS one [207]. In conclusion, this highlights that the era of CPs is still in an early phase regarding biomedical applications, and more mechanistic research is required with these promising materials in bioelectronic device applications to move toward clinical translation.

### 6.1. Implantable Devices for Electrical Recording

An implantable sensor for neural recording was developed for both brain and spinal cord applications in mice using a flexible PEDOT:PSS–polyurethane PDMS device. This device demonstrated high flexibility, stretchability, and biocompatibility. Additionally, at neuronally relevant frequency ranges, the PEDOT:PSS device showed lower impedance compared to a traditional metal platinum electrode. This resulted in higher SNR values and thus improved neural recording quality [208].

In another example, an implanted CP MEA capable of both recording and stimulating specific brain regions with a high resolution was demonstrated due to the low impedance of the device. When combined with MRI, these devices provide spatial coverage of brain activity, allowing the detection of single-neuron APs due to the high SNR of the electrodes. The described MEA was composed of PEDOT:PSS-coated gold electrodes, encapsulated in Parylene C, ensuring its flexibility and conformability [209].

### 6.2. Wearable Devices for Electrical Recording

A wearable recording device was developed using a multicomponent hydrogel containing PEDOT:PSS. The device was designed to detect the temperature and strain during patient movement, allowing the health monitoring of patients with arthritis or those recovering from joint diseases. In addition, in further in vitro testing, the hydrogel showed excellent cytocompatibility, maintaining over 90% cell viability after three days. In a subsequent study, the device was also proven to be effective in capturing electrophysiological signals, such as electromyography, with higher sensitivity compared to commercial Ag/AgCl electrodes [210].

Another example of a wearable recording platform was demonstrated an elastic OECT using gelatin as a flexible substrate and PEDOT:PSS as the CP for the source and drain electrodes and the channel of the transistor. This platform delivered high transconductance (12.7 mS) while showing durability and superior elastic properties, which are important features for devices attached to the skin. It showed slower, synapse-like current recovery after a voltage spike and improved biosensing functions. Additionally, the gelatin used in the device could be recycled without compromising its performance [211].

### 6.3. Implantable Devices for Electrical Stimulation

Implantable devices are currently being explored for the electrical activation of nerve tissue in rodent models to promote nerve repair and regeneration. To improve the interface with the biological system, a PEDOT:PSS electrode can be embedded in various hydrogels, which provide key components. For example, phenyl-borate-conjugated alginate could enhance the adhesion and conformability to the nerve, while methacrylate-conjugated alginate ensures elasticity. It has been shown that a tissue recovery improvement after short-term stimulation can be achieved, together with degradability, which eliminates the need for a second surgery for the removal of the device [205].

Another interesting implantable stimulation platform was developed in a mouse model [212]. Here, an electrode was implanted in the primary somatosensory cortex, a region that is crucial for movement, which can be triggered by olfactory stimuli. The device, composed of PDMS/PPy/Cu, detects specific smells through a surface–triboelectric coupling effect and stimulates the cortex, inducing movement to “avoid danger”, which can be extremely important in fleeing from predators or seeking safety. The sensor then detects this movement and stimulates the brain again, enabling a closed-loop feedback system. This in vivo platform could be further developed for patients with sensory impairments to help restore lost functions.

A study using CPs demonstrated vision restoration in a rat model of retinal dystrophy. This “liquid retina device” approach utilized P3HT nanoparticles as light-responsive stimulators, activating retinal neurons that were non-functional in these rats. Unlike traditional prosthetic devices, the injection of these nanoparticles allowed them to spread throughout the subretinal space, enabling neuronal stimulation without triggering inflammation. This breakthrough holds significant potential in enhancing the spatial resolution, which is lacking in current retinal prostheses [213].

Neuronal activity stimulation was also demonstrated in another study targeting the peripheral sciatic nerve in rats. The implanted device was a photocapacitor presenting a p-n electrode composed of CPs, with N,N′-dimethyl perylenetetracarboxylic bisimide (PTCDI) as the n-type material and phthalocyanine (H2Pc) as the p-type material. By detecting deep-red light, the device enabled wireless nerve stimulation while maintaining flexibility, minimizing tissue damage, and ensuring stable positioning for direct nerve activation [214].

### 6.4. Wearable Devices for Electrical Stimulation

Another recent technology used temporary tattoo electrodes, offering good skin conformability for EEG testing. The device was designed as an all-polymer PEDOT:PSS printed electrode and was compared with an Ag/AgCl standard electrode used for EEG. A complementary skin-mimicking in vitro platform was developed, composed of a stimulation-recording unit, where the two types of recording electrodes (CP and standard electrode) were compared with a Cu electrode used for stimulation. The CP electrode provided high-quality recordings and greater precision regarding neuronal activity due to its physical properties [215,216].

While organic bioelectronics have shown great promise in vivo, further research and development are essential to fully realize their potential and bring these devices toward clinical application. With continued progress, these organic materials offer exciting possibilities in advancing medical treatments and improving patient outcomes.

## 7. In Vitro Applications

Animal models, mainly rodents, are the current gold standard in biomedical research. They provide insights into how biological systems interact in a whole-organism context and provide a controlled alternative to human studies under strict regulatory oversight. According to the Nuremberg Code, human experiments should be “designed and based on the results of animal experimentation”, making animal experimentation an obligatory step in many cases. While animal models are still required for the initial scientific and safety validation of preclinical therapeutics, the FDA Modernization Act 2.0 recognizes its limitations in accurately modeling the human physiology and empowers researchers to employ innovative non-animal models [217]. Indeed, animal models are, in many aspects, poor predictors of human disease, leading to different outcomes between animal studies and clinical trials and resulting in poorly targeted therapies for the general patient population [218]. Moreover, a new movement emerged 65 years ago with Russel and Burch introducing the 3Rs principle: Replace, Reduce, and Refine. Their aim was to ensure the humane and responsible use of animals in scientific studies. This principle has become a cornerstone of ethical animal research worldwide and has been embedded in current regulations and guidelines, such as those from the European Union and National Institutes of Health. 

In vitro human systems are emerging as alternatives to animal models that offer the ability to study specific cellular and tissue processes using human cells or tissues. These laboratory methods are typically designed to replicate certain aspects of the human physiology. Unlike in vivo systems, which study processes within the complex environment of a living organism, in vitro systems offer a controlled and simplified setting to replicate the human physiology and mechanisms in a defined environment. 

In vitro systems offer numerous advantages compared to in vivo ones: (i) cost-effectiveness, avoiding the high economic and time costs of animal research; (ii) targeted variable testing, allowing researchers to focus on specific variables without interference from systemic factors such as hormonal, immune, or environmental influences; (iii) higher throughput, enabling rapid hypothesis testing or drug screening; (iv) ethical benefits, by reducing the reliance on animal testing; (v) dynamic monitoring, allowing the easy integration of technologies to monitor biological systems at a high resolution. 

These models encompass a variety of methods tailored to studying specific biological processes or functions. The initially used 2D systems provided a methodology to address specific questions with limited complexity but lacked the 3D environment that cells face in vivo. As a result, 3D models have been developed to create a more biomimetic environment and study cellular responses to biophysical factors. However, their simplified nature can be a limitation, as they may not fully replicate the intricate interactions found in living organisms, leading to challenges when extrapolating the results to in vivo conditions. 

To address these limitations, a new generation of in vitro models has been developed: MPSs. MPSs are generally defined as in vitro platforms composed of cells or tissues of human or animal origin, exposed to a microenvironment designed to mimic the physiological aspects of tissue and organ function [219]. These systems are typically designed to recapitulate key properties of human organs or tissues (e.g., physical, biochemical, electrical, mechanical, structural, morphological) and mimic their primary functions as dictated by the research questions, seeking to better reflect the human physiology under healthy and pathological conditions. MPSs are micro-engineered devices that combine biomaterials, 3D cell cultures, and microfluidics [220]. Their microscale dimensions and integration of microfluidic technology ensure low waste generation, high accuracy, and high throughput. MPSs include, but are not limited to, organ-on-chip systems, organoids, and 3D co-cultures with matrices or 3D bioprinted tissues. By offering clinically relevant tools to study disease mechanisms, develop diagnostics, and screen therapeutic drug candidates, MPSs hold significant potential in improving our understanding of human pathophysiology and accelerating the development of targeted and reliable therapies. 

CPs have emerged as pivotal materials in MPSs due to their ability to interface with biological systems under physiological and dynamic settings, providing label-free, non-invasive, and real-time multiparametric information on cellular behavior and interactions at the single-cell level [9]. Their integration enables applications such as electrical stimulation, real-time electrical monitoring, detailed cellular function and tight junction monitoring, and biosensing. 

### 7.1. Electrical Stimulation 

Electrical stimulation in MPSs is employed to recreate physiological environments and to promote tissue development or homeostasis. CPs such as PEDOT:PSS enable precise and tunable stimulation. For instance, a study demonstrated the use of a stretchable mesh electrode system for the monitoring and modulation of the electrical activity of human cortical organoids. The 50-µm-diameter and 5-µm-thick electrodes, composed of a stretchable PEDOT:PSS hydrogel encapsulated in poly(styrene ethylene butylene styrene), maintained stable electrochemical impedance under 50% compressive or tensile strain. This system, seamlessly integrated with the organoids’ 3D structures for more than three months, successfully delivered electrical stimulation to the organoids (100 pulses and 100 Hz at 100 µA and 20 µA), affecting the calcium response, thus demonstrating its potential as a long-term electrophysiological interface [221].

Similarly, a heart-on-chip platform with gelatin–PEDOT:PSS pillars, replicating cardiac tissue’s mechanical properties, was developed. The short-term gradual electrical stimulation of immature cardiomyocytes over one week (from 2 Hz to 6 Hz, with a step-up stimulation of 1 Hz every day) induced spontaneous contractions of the cells and significant cytoskeletal changes, resulting in sarcomere anisotropy, increased connexin-43 expression, and directional alignment [222]. 

### 7.2. Electrical Monitoring 

While electrical stimulation is essential in eliciting physiological responses in MPSs, its full potential is exploited when paired with real-time electrical monitoring, which captures the induced responses and provides feedback on tissue or cellular behavior. 

In addition to electrical stimulation, the previous heart-on-chip platform included PEDOT:PSS pillars to perform the non-invasive electrophysiological monitoring of the cardiac tissue. This dual approach allowed the researchers to perform the real-time recording of the tissue’s AP propagation activity as an extracellular field potential, correlating the stimulation with electrical activity. Rhythmic peaks with minimal noise were observed due to the higher performance of the PEDOT:PSS hydrogel [222]. 

Another study developed vertical and flexible micropillar microelectrodes for the non-invasive recording of extracellular field potentials in cardiac tissue. PEDOT:PSS micropillars were 3D-printed onto 36 gold microelectrodes in a 6 × 6 array format and embedded within a cell-laden hydrogel. The micropillars successfully characterized the tissue’s electrical properties with and without epinephrine as a model drug, with minimal interference due to their flexibility and high aspect ratio [223]. 

Furthermore, the two preceding research studies combined cellular activity monitoring with the precise measurement of the tissue’s contractile forces. This was performed through the optical tracking of the PEDOT:PSS pillars’ displacement [222] or by adding a thermoplastic elastomer and quantum dot microwires, acting as contractility sensors [223]. This demonstrates the potential to add multiple functionalities to a single device. Similarly, the integration of a PEDOT:PSS-coated MEA into a stretchable PDMS membrane allowed for the incorporation of additional functionalities—namely, the ability to apply mechanical stimulation while performing electrical monitoring. This added capability enhances the system’s ability to study the dynamic interaction between electrical signals and mechanical forces, providing deeper insights into cellular behavior under both mechanical and electrical influences [224]. 

Building on the application of electrical monitoring in 3D cell cultures, recent advances have extended these principles to more complex structures, such as organoids. With their higher level of physiological complexity, organoids pose unique challenges and opportunities for sensor integration, necessitating innovative approaches to ensure accurate and reliable monitoring. 

The integration of CPs into organoids has been extensively explored. For example, a transparent 3D wafer-integrated MEA shell with three tunable self-folding leaflets was used to enclose brain organoids. Each leaflet featured a PEDOT:PSS-coated electrode at its extremity, enabling the electrophysiological recording of organoids ranging from 400 to 600 µm in size. The PEDOT:PSS coating reduced the electrode impedance and minimized the Young’s modulus mismatch, improving the electrode–organoid interface. While the number of electrodes was limited, this system recorded spontaneous field potentials in brain organoids for up to four weeks and captured responses to glutamate stimulation while allowing bright-field and fluorescence imaging [225]. 

Furthermore, the number of electrodes has been increased to develop a self-rolled biosensor array of passive MEAs for the electrophysiological recording of cardiac spheroids’ field potentials, enhancing the resolution of the electrical monitoring system. The array’s controlled geometry wraps around spheroids of 50 to 200 µm in diameter, positioning electrodes uniformly around it. The reduced electrode impedance due to PEDOT:PSS electrodeposition (from 0.56 ± 0.25 Mohm to 14 ± 7.6 kohm at 1 kHz) and the improved integration method enhanced the recording quality, enabling the evaluation of electrical signal propagation in 3D [226]. 

Taking this development further, a liquid metal–polymer conductor-based mesh with a 128-channel MEA on a compact 2 × 2 mm surface was introduced to interface with human hippocampal organoids. This deformable and highly stretchable (up to 500%) mesh could be positioned on the upper and lower hemispheres of the organoid, enabling the recording of spontaneous neural spikes, synchronization, and oscillatory activity. Each electrode captured multiple signals, displaying two to four distinct spike types after sorting. PEDOT:PSS electrodeposition improved the device’s stability by preventing gallium indium alloy degradation, therefore ensuring the long-term integration of the device [227]. 

These three studies illustrate the progressive integration of electrical monitoring systems with organoids, from the initial use of a small number of electrodes to advanced high-density arrays capable of providing detailed spatial and temporal information. As polymer-based electrode arrays become more complex (e.g., increased number of electrodes, reduced electrode impedance, better stability) and better integrated with biology, the ability to monitor intricate cellular dynamics will be improved, opening up new possibilities for more accurate and versatile organic bioelectronic applications. 

### 7.3. Cellular Function and Tight Junction Monitoring 

Monitoring cellular function and tissue behavior is central to understanding biological processes in MPSs. CPs facilitate this by providing both structural support and electrical cues. For instance, PEDOT:PSS electrodes were integrated into an organ-on-chip to track the intestinal barrier’s formation, disruption, and recovery while allowing optical inspection. The microfluidic chip, fabricated from a biocompatible and transparent cyclo-olefin polymer, incorporated a semipermeable PET membrane that supported human intestinal colorectal adenocarcinoma cells. This membrane was positioned between two low-polarization and semi-transparent PEDOT:PSS electrodes patterned on Ti/Au ring-shaped structures. This study reported uniform current distribution across the entire cell culture area, allowing the successful monitoring of the cell junction strength, intercellular cleft morphology, and cell surface area in real time over one week [54]. 

While the initial approach employed cells on a PET membrane between CP-based electrodes to monitor the intestinal barrier’s integrity, this can be taken a step further. Indeed, intestinal cells have been integrated within a tubular luminal PEDOT:PSS scaffold that not only replicated the structural features of the gut but also incorporated electrical monitoring capabilities. This scaffold, employed as a 3D template to support a human gut epithelium model, facilitated the development and maintenance of a functional intestinal epithelium, characterized by a stratified and polarized epithelium lining the scaffold lumen, anchored to a lamina propria-like tissue. The researchers reported tissue formation monitoring, including cell presence, adhesion, and growth monitoring, over 26 days using EIS and transconductance measurements. This revealed distinct electrical signatures corresponding to various cell and tissue types [228]. This advancement demonstrates a significant leap in device design, merging structural biomimicry with functional monitoring in a single platform. 

### 7.4. Biosensing 

Electrical monitoring and stimulation provide critical insights into the dynamic activities of cells, offering a direct window into the physiological state of the system. Integrating biosensors into MPSs enhances this capability by enabling the real-time, in-line, and non-invasive detection of key functions, biomolecules, and signaling events driving cellular behavior. This integration does not only enable us to improve our understanding of biological processes or events, but also paves the way for more advanced and functional MPS platforms with dynamic and high-resolution monitoring, which, at present, is not possible in vivo [229]. With their high sensitivity, redox activity, and electrical conductivity, CPs are particularly well suited for this purpose, playing an essential role in the biosensor field. 

As an example, a micron-scale OECT device was integrated with a microfluidic platform for the label-free detection of Alzheimer’s biomarker Aβ aggregates in human serum. The OECT channel, composed of PEDOT:PSS (depletion mode) or p(g0T2-g6T2) (accumulation mode), was separated from the electrolytes by a nanoporous membrane functionalized with Congo red molecules, which specifically bind Aβ aggregates, thus altering the transistor’s characteristics. The authors demonstrated the successful detection of Aβ aggregates across an eight-orders-of-magnitude concentration range (2.21 fM to 221 nM) in just 1 µL of human serum [230]. 

PEDOT:PSS-based OECT microarrays were also used for the selective sensing of K^+^ and Na^+^ concentrations. For this, a 7 × 7 mm^2^ chip featuring 16 individually addressable gold interdigitated electrodes coated with PEDOT:PSS was developed and integrated with ion-selective membranes for targeted ion detection and with a microfluidic cell for continuous perfusion. Ion concentrations ranging from 1 to 100 mM were monitored by measuring the drain currents of the OECTs with buffer solutions of varying concentrations. The device demonstrated good selectivity and sensitivity, influenced by the ion-selective membrane’s thickness. They observed that a reduction in membrane thickness led to an increase in sensitivity and decrease in selectivity. The system enabled the multiplexed, real-time, and reversible sensing of Na^+^ and K^+^ concentrations, highlighting its potential for integration into more complex microfluidic platforms [37]. 

Another study reports the development of an electrochemical sensor to detect α-synuclein, a presynaptic neuronal protein implicated in Parkinson’s disease at high concentrations. PEDOT-OH nanotubes were synthetically self-assembled using a peptide sequence from α-synuclein as a template molecule, creating a peptide-imprinted electrically CP on indium tin oxide electrodes. The resulting poly(PEDOT-OH) formed tubular structures with an imprinting factor of ca. 2.5 at 0.65 fM, demonstrating high selectivity and affinity for α-synuclein. The peptide-imprinted electrodes detected the α-synuclein concentration in real time, ranging from 0.065 pM to 65 nM, in a brain organoid culture medium, making the sensor suitable for 1000-fold diluted cerebrospinal fluid samples [231]. 

Biosensing has emerged as a powerful tool in MPSs, enabling the real-time and quantitative monitoring of key biomolecules. The integration of CPs offers high sensitivity, stability, and seamless integration with MPS platforms. Looking forward, advancements in sensor miniaturization, multiplexing, and coupling with other monitoring modalities, such as electrical and mechanical sensing, will further expand the utility of electrochemical sensing. 

Significant progress has been made in integrating CPs into MPSs; however, challenges persist. One major hurdle lies in ensuring a delicate balance between maintaining high electrical conductivity, biocompatibility, and suitable mechanical properties. Another key challenge is the seamless integration of CPs into complex MPS platforms without compromising their functionality or structural integrity. Additionally, the fabrication process demands high precision and scalability, particularly when working with soft materials like hydrogels. Customization for specific applications adds another layer of complexity, as the CPs’ properties (e.g., conductivity or mechanical flexibility) need to match the specific requirements of the studied cells, tissues, or organs, which often requires significant resources and iterative design. Addressing these challenges will be pivotal to unlock CPs’ full potential in biomedical research and applications. 

To pave the way for more advanced and versatile MPS applications, future efforts should focus on (i) developing robust and tunable materials, (ii) improving the microfabrication and integration techniques, (iii) developing sensors capable of the multimodal detection of multiple biochemical and electrical signals, (iv) making these systems scalable and cost-effective, and (v) employing advanced computational tools to interpret complex datasets and therefore bridge the gap between electrical signals and biological phenomena. 

## 8. Conclusions and Future Visions

In this review, we explored the application of organic bioelectronics to study, characterize, and stimulate biological processes, with a focus on in vitro MPS systems, while including the current state of clinical and in vivo applications. Specifically, we demonstrated their capacity to dynamically transduce a broad spectrum of biological events relevant for human physiology. It is clear that CPs represent a versatile and biocompatible class of materials for interfacing with biological systems, providing functional alternatives to conventional inorganic materials and emerging 2D nanomaterials like graphene and MXenes.

Unlike metals, which are rigid, CPs are soft and flexible, enabling improved integration with biological systems. Compared to graphene and MXenes, which require additional biofunctionalization and primarily rely on electronic conduction, CPs offer intrinsic biocompatibility, tunable electrochemical properties, and mixed ionic–electronic conduction, allowing for the seamless transduction of biological events and making them more effective for bioelectronic applications.

Their ease of processing allows the fabrication of various devices—MEAs, electrolyte-gated transistors, ion pumps, and photoactuators—facilitating their integration with biology with reduced mechanical mismatching while conforming to curved or dynamic biological tissues. Additionally, their mixed conduction enhances signal detection, making them particularly suitable for applications that require high sensitivity and spatial resolutions. Beyond these advantages, their capacity for dynamic and active sensing, efficient operation in environments replicating interstitial tissue, transparency for seamless integration with optical imaging, and low-voltage operations make them particularly well suited for a wide range of life science applications.

As the field of organic bioelectronics expands, there is growing recognition that the integration of sensors with biological systems is crucial in obtaining robust, reliable, and reproductible data. While significant progress has been made, we are still in the early stages of incorporating these materials into MPSs, and further advancements are required. Many challenges still lie ahead that need to be satisfied before the technology reaches a level of maturity that will allow clinical translation and market entry. These challenges span from requirements in material optimization to the need for the realization of novel device architectures and from demands for extended operational stability to prerequisites for seamless integration into the current industrial ecosystem.

One of the major criticisms of organics concerns their long-term stability in real physiological conditions. Bioelectronic devices should ideally be able to operate for extended time periods, ideally for years, without compromising their performance. Although, in the past, academic publications did not focus specifically on presenting devices with long-term stability, multiple groups have recently started reporting organic electronic devices with remarkable reproducibility and stability, which, in some cases, can reach up to a few years [232]. In parallel, synthetic chemists are exploring new ways to produce materials with improved long-term stability by designing molecules with optimized structures for the reliable uptake and release of ions, by balancing their energy levels at a molecular level, and by altering their molecular structures or optimizing their post-processing treatments to produce new and stable organic materials [233,234]. The organic electronics community today understands the importance of device longevity, and steps are being taken daily toward more stable and reliable organic devices.

Another challenge for organic electronics is their scalability and their integration into the existing industrial infrastructure. Organic materials are fully compatible with additive manufacturing techniques [235], which means that they can participate in the upcoming manufacturing revolution (Industry 4.0), which is largely linked to 3D printing technologies [236]. Organic materials are also compatible with photolithography techniques, which support the high-yield manufacturing of devices with large numbers of electrodes and transistors integrated in small spaces and with low spatial variability—features that are extremely important in bioelectronics. Therefore, high-density organic electronic MEAs and arrays of transistors have already been presented [237]. Of potential concern are the challenges that organic materials face at the high temperatures needed during the back-end-of-line (BEOL) processes associated with CMOS technologies. Nevertheless, the use of blend composites, which results in thermally stable CPs [238], especially when complemented with molecular redesigning, seems to be a promising approach for the realization of inorganic/organic hybrid technologies. These technologies envision the best of both worlds, with the advantages of organic materials at the interface between biology and technology to be supported by the monolithic integration, high density, and processing power that CMOS technology offers. Furthermore, the proven ability of organics to support integration with microfluidics [239] strengthens the potential of organics in the world of microphysiological systems.

In the near future, organic devices are expected to fully enter the clinical setting. In order to do this, they will need to be approved by regulatory authorities after undergoing clinical and pre-clinical trials, like any other medical device. These pre-clinical trials will be used to address any potential safety concerns before the devices are used in humans. These clinical trials can be categorized as phase I–IV, where phase I provides initial testing in humans, primarily to test their safety in a small number of patients; if successful, each of the following phases increases the number of included patients and the protocol for testing. This detailed and closely regulated clinical testing ensures that the device’s safety profile and its potential risks are evaluated, as well as its effectiveness, progressing to post-market trials, which involve larger sample sizes and comparisons to established treatments. The bioelectronic community can capitalize on the already gained experience from existing biomedical technologies in order to facilitate their fast adoption in clinical environments.

In terms of the vision of CPs as an interface between biological tissues and electronics for in vitro modeling applications, the integration of AI and machine learning (ML) holds transformative potential in enhancing the precision, efficiency, and adaptability of biomedical research [240,241]. AI-driven computational modeling can further accelerate the discovery and optimization of novel polymer formulations by predicting their electrical, mechanical, and biocompatibility properties, reducing the reliance on trial-and-error approaches, and improving their long-term stability. ML algorithms can further enhance the real-time sensing capabilities by improving signal processing, pattern recognition, and adaptive feedback loops within the in vitro model system for the monitoring and prediction of cellular signaling interactions, including MPSs. This would enable the more accurate and dynamic monitoring of biochemical and physiological changes, specifically when studying cells with weak signals. As AI continues to evolve, its synergy with CP research will pave the way for smarter, more responsive biosensors, ultimately improving disease modeling, drug screening, and personalized medicine applications.

Future developments require a focus on several key aspects. First, expanding the use of CPs to transduce signals from non-electrogenic cells represents a promising direction. While electrogenic cells have been extensively studied due to their well-defined electrical activity, recent research highlights the role of the resting membrane potential and ion channels in non-excitable cells, opening up new opportunities for bioelectronic sensing. Second, multimodal devices that combine stimulation and real-time monitoring would streamline experiments, reducing their complexity and enhancing the research efficiency. Additionally, integrating CPs into tissue engineering scaffolds or hydrogels could provide structural support and active bioelectronic functionalities. Finally, scalability remains a critical challenge as significant efforts are needed to bring organic bioelectronics to a high level of reliability and manufacturability.

Through continued innovation, the full potential of organic bioelectronics will be fully exploited, driving the next generation of bioelectronics.

## Figures and Tables

**Table 1:** Conductivity, mechanical properties, and biocompatibility of current and promising conjugated conducting polymers used for biomedical applications.

Polymer Name	Abbreviation	Type of Doping	Electrical Conductivity	Young’s Modulus	Biocompatibility
Poly(3,4-ethylenedioxythiophene): poly(styrene sulfonate)	PEDOT:PSS	p	0.1–1 to 4380 S/cm [86,87]	Hydrogels: 2–10 MPa [84]; films: 0.9 ± 0.2 to 2.9 ± 0.5 GPa at 55% and 23% relative humidity [94]	High biocompatibility in printing inks after collagen coating [88] or in scaffolds [89]
Polypyrrole	PPy	p	7.5 × 10^3^ to 10 × 10^3^ S/cm [95,96]	180 MPa to 4.98 GPa [97,98]	Partial and concentration-dependent toxicity [99]. Structure [100], coating, and surface modification [101] can improve the biocompatibility
Polyaniline	PANI	n, p	10^−10^ to 10 S/cm for standard doped PANI [102]; 30 to 200 S/cm for PANI with strong protonic acid doping [95,103]	2.9–3.1 ± 0.2 GPa [104]	Significant cytotoxicity due to residual ammonium persulfate and low-molecular-weight polar substances [105]
Poly(3-hexylthiophene-2,5-diyl)	P3HT	p	10^−4^ to 224 S/cm [106]	260 ± 27 MPa [107]	Stable in physiological media but limited biocompatibility. Can be improved by introducing cell adhesion functional groups through protein-based coating and by manipulating the surface wettability [108,109]
Poly(2-(3,3′-bis(2-(2-(2-methoxyethoxy)-ethoxy)ethoxy)-[2,2′-bithiophen]-5-yl)thieno[3,2-b] thiophene)	p(g2T-TT)	p	1 to 616.7 S/cm [110,111]	N/A	No toxicity when exposed to tissues [112]
Poly((ethoxy)ethyl2-(2-(2-methoxy ethoxy)ethoxy)acetate)-naphthalene-1,4,5,8 tetracarboxylic-diimide-co-3,3ʹ-bis(2-(2-(2-methoxy ethoxy)ethoxy)ethoxy)-(bithiophene))	p(gNDI-T2)	n	0.1085 S/cm; shows the highest capacitance of the channel per unit volume (397 F.cm^−3^) and the smallest electronic carrier mobility (0.00031 cm^2^/V/s) among other p-type polymers [113]	N/A	N/A

## Data Availability

Not applicable.

## Supplementary Materials

The following supporting information can be downloaded at https://www.mdpi.com/article/10.3390/bios15040253/s1, Table S1. Overview of conducting polymers’ properties; Table S2. Overview of current clinical trials on conducting polymers.
